# Analysis of lipid-assisted self-assembly of hydrophobic CuInS_2_/ZnS quantum dots into water-stable nanoclusters that perform intra-cluster energy transfer

**DOI:** 10.1039/d6nr00297h

**Published:** 2026-05-20

**Authors:** Joel T. Whipp, Ashley M. Hancock, Zabeada Aslam, Kevin Critchley, Peter G. Adams

**Affiliations:** a School of Physics and Astronomy, University of Leeds Leeds LS2 9JT UK k.critchley@leeds.ac.uk p.g.adams@leeds.ac.uk; b Astbury Centre for Structural Molecular Biology, University of Leeds Leeds LS2 9JT UK; c Leeds Electron Microscopy and Spectroscopy Centre, School of Chemical and Process Engineering, University of Leeds Leeds LS2 9JT UK

## Abstract

Quantum dots (QDs) are nanocrystalline semiconductors that have the ability to perform efficient Förster resonance energy transfer (FRET) to biomolecules and other nanoparticles. Aqueous environments are required for applications related to biological systems, and common hydrophobic QDs will tend to cluster under these conditions. Whilst large (microscale) aggregates of QDs are undesirable, small (nanoscale) clusters could have applications as the active component in bio-imaging, bio-sensors or bio-photovoltaics. In this study, we developed a new procedure to utilize lipids to control the assembly of ‘nanoclusters’ of copper indium sulfide/zinc sulfide (CuInS_2_/ZnS) core/shell QDs. High-resolution electron microscopy revealed that the QDs had a particle size of ∼3.3 nm when they were in organic solvents and formed clusters of ∼40 nm when the nanoparticles were exchanged into aqueous solution in the presence of lipids. Particle sizing by nanoparticle tracking analysis suggested that the overall hydrodynamic size was 100–200 nm, which is consistent with an electron-dense core of clustered QDs surrounded by looser lipid assemblies or higher-order structures. The lipid was found to be essential for stabilizing the cluster, with fluorescence microscopy and spectroscopy confirming that the lipids and QDs were colocalized (using fluorescently-tagged lipids). Ensemble spectroscopy measurements revealed that there was a consistent red-shift of the emission peak, and a reduction in the excited state lifetime, for QD nanoclusters suspended in an aqueous buffer as compared to isolated QDs dissolved in organic solvents. The combined data provides strong evidence that downhill energy transfer occurs within one cluster from small, high-bandgap QDs to larger, low-bandgap QDs. Our findings contribute to a new understanding of how QD–QD and QD–lipid interactions influence their photophysical properties. Our work provides a new protocol based on a physical self-assembly that is modular and adaptable: alternative lipids or QDs and different buffer conditions (salt, pH) could be used. Future work could incorporate membrane proteins in the development of QD-biohybrid systems towards new applications in bio-nanotechnology.

## Abstract 2

A procedure was developed to assemble hydrophobic quantum dots and lipids, producing nanoclusters that are stable in aqueous media. The structural change was associated with a fluorescence red-shift and quenching caused by QD-to-QD energy transfer.

## Graphical abstract

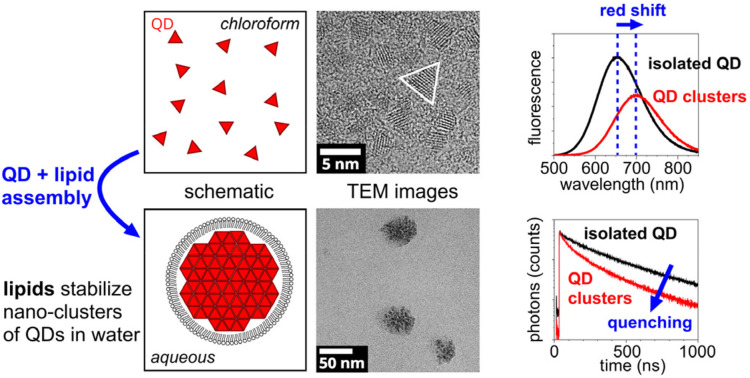

## 1 Introduction

Quantum dots (QDs) are semiconductor nanocrystals that exhibit remarkable optical properties, including size-tunable fluorescence, high quantum yields, and exceptional photostability. These features have enabled their widespread use in a range of technologies, from solar energy conversion and biosensing to commercial display systems.^1–16^ As interest in QDs has expanded beyond fundamental studies into real-world applications, advances in surface functionalization and colloidal stabilization have become increasingly important, particularly for their integration into biological environments. Among the various QD compositions, CuInS_2_/ZnS nanocrystals have gained attention as a cadmium-free alternative with lower toxicity and strong optical performance.^17–22^ These QDs have been widely studied with respect to their excited-state dynamics, recombination pathways, and the relationships between structural characteristics and optical properties such as emission wavelength and extinction coefficients.^23–31^ The addition of a ZnS shell can enhance the photoluminescence of CuInS_2_ QDs by approximately 40-fold, resulting in a blue shift of the photoluminescence spectrum. This phenomenon arises from the formation of a semi-alloyed CuInS_2_/ZnS particle rather than a conventional core/shell structure with a well-defined boundary, due to cationic exchange between CuInS_2_ and ZnS lattices.^32^ CuInS_2_/ZnS QDs exhibit broad excitation and emission spectra, long fluorescence lifetimes, and a substantial Stokes shift in comparison to CdSe QDs.^33^ These exceptional optical properties position CuInS_2_/ZnS QDs as ideal components in bio-hybrid nanomaterials, allowing for the synergy of optimized biological macromolecules with custom-designed nanoparticles to create highly efficient ‘nanocomposite’ light-harvesting materials.^34–36^

In optoelectronic devices, such as light-emitting diodes (LEDs), solar cells, and biosensors, Förster resonance energy transfer (FRET) between QDs plays a significant role. In QD photovoltaics, FRET usually results in energy loss, making it undesirable.^37^ A similar scenario arises in light-emitting devices utilizing core-only QDs, where energy may be transferred to non-luminescent QDs, leading to non-radiative recombination.^38,39^ Conversely, in core–shell QD arrays, FRET can facilitate the migration of excited states, enabling high-brightness QD LED devices that would otherwise suffer from poor charge injection.^9^ Pioneering investigations into FRET among dried QDs postulated that FRET occurs where there is an inhomogeneous size distribution of QDs, specifically the transfer of energy from relatively small, high energy QDs to larger, lower-energy QDs, when these QDs are co-localized (near to each other).^40,41^ Subsequent experiments examined CdSe QDs that had defined sizes (and therefore energies) deposited in layers, where energy transfer was observed from a “monodisperse blue” QD layer to a “monodisperse red” QD layer, across an energy difference of tens of milli-electronvolts.^42^ A rapid nanosecond decay in fluorescence due to FRET was noted when exciting high-energy “blue” QDs, with the QD excited state lifetime (*i.e.*, fluorescence lifetime) showing size-dependent behavior, increasing in “redder” QDs that primarily acted as energy acceptors (timescales of roughly 2–20 ns). Similar findings were observed with CdTe QDs that were assembled into layers, resulting in a red-shift in the photoluminescence peak as compared to individual QDs.^43^ Fast energy transfer (approx. 250 ps) was observed, supporting the notion of FRET from smaller QDs (high-energy) to larger QDs (low-energy). Investigations of CuInS_2_/ZnS QDs deposited as a thin film again revealed a red-shift in fluorescence peak attributed to energy transfer from smaller to larger QDs.^44^ Exciton hopping between QDs within arrays has been modelled using computational simulations.^45^ Overall, it is clear that wherever QDs are tightly associated there will be FRET between the individual QD particles, in a direction from high- to low-energy states.

Water-solubilized “clusters” of QDs^46–49^ and metal nanoparticles^50–54^ can be useful for their compatibility with biological environments, allowing applications in bio-imaging or biosensors. Notably, these clusters of QDs have modified optical properties compared to individual QDs, such as higher brightness, and they can participate in FRET, as described above for solid films of QDs. However, many QDs have surface chemistry that makes them hydrophobic and therefore challenging to use in aqueous suspensions, where these QDs exhibit instability unless they are encapsulated by a more hydrophilic or amphiphilic material. If placed in aqueous solvent, hydrophobic QDs are likely to spontaneously self-assemble into clusters of several particles, contingent on the physicochemical conditions to which they are exposed, including the ionic strength of the solvent, the temperature, pH and the surface chemistry of the QDs.^46–48^ Studies of water-solubilized QD nanoclusters can be compared with the numerous studies of QD clusters, aggregates and superlattices that can be assembled in organic solvents.^49,55–58^ And studies of clustered QDs can be contrasted with other types of nanoparticles, for example, a FRET system based on silver nanoparticles combined with silicon nanoparticles into clusters was stable in aqueous environments and also exhibited efficient energy transfer.^52^ QD clusters can vary widely in size, ranging from micro-scale “crystallites” containing many thousands of QDs^49,55^ to relatively small nanoscale clusters consisting of 10–100 QDs.^46,47^ Despite significant work on the photophysical properties of QD aggregates, it remains challenging to control the overall size of the cluster of QDs. It would be useful if there was a clear methodology for assembling QDs into clusters, with a robust means of stabilizing the outer edge of the cluster, in order to allow control over the overall cluster size.

We can look towards biomolecules that are naturally soluble in water when considering how to stabilize nanoparticles in the same environment. Previous studies have shown that nanoparticles can be stabilized in aqueous environments by attaching suitable hydrophilic biomolecules to the exterior surface of the nanoparticle, such as small proteins^59^ or reactive amino acids.^54^ Lipids, the biomolecules that form cell membranes,^60^ could be used to provide a stable coating around clusters of nanoparticles to make them compatible with aqueous environments. The concept of utilizing lipids for nanoparticle stabilization can make use of the known property of lipids to self-assemble into robust monolayers or bilayers in water.^60^ Lipids have been demonstrated to engage with hydrophobic nanoparticles in a few prior studies.^61–68^ For example, the direct covalent attachment of lipids to the exterior surface of iron oxide nanoparticles was successful in stabilizing individual nanoparticles in aqueous solutions.^61^ Alternatively, the non-covalent assembly of lipids around (16 nm) CdSe/ZnS QDs yielded 100 nm clusters that were also stable in water.^62^ There, fluorescent NBD lipids functioned as probes for the activity of an enzyme, phospholipase A_2_, whereby the NBD fluorescence was initially quenched by the QD cluster but the emission greatly increased upon lipid digestion by the enzyme.^62^ The exploration of other types of lipid/nanoparticle assemblies has extended to the embedding of small hydrophobic nanoparticles within the hydrophobic core of the lipid bilayer of vesicles,^64–66^ presenting a prospective avenue for establishing a stable, enclosed environment conducive to FRET interactions between QDs. These previous studies have shown the potential for QDs associated with lipids. Many excellent studies have developed complex systems involving Cd-based QDs but fewer have used CuInS_2_ QDs, as cited. It is timely to increase our exploration of alternative QDs that are more sustainable/less toxic. The current study focuses on developing a new procedure that exploits the self-assembly of lipids to stabilize “nanocluster” structures of CuInS_2_/ZnS QDs that are stable in aqueous solutions. We aimed to characterize the size and structure of these assemblies and to comprehend and compare the decay and energy transfer mechanisms of the individual *versus* the clustered form of these QDs. A key contribution is our demonstration that lipids can be effective in controlling the size and stability of the QD cluster with a simple self-assembly protocol. It is valuable to know that this procedure works with CuInS_2_/ZnS QDs that have much lower toxicity than Cd-based QDs. Our results are also significant for providing a comprehensive structural and optical explanation of this multi-component system.

## 2 Experimental section

### 2.1 Materials

Indium(iii) acetate (99.99%), copper(i) iodide (99.999%), octanethiol (≥95%), zinc stearate (90%) and octadecene (ODE, 90%) were purchased from Sigma Aldrich. 1,2-Dioleoyl-*sn*-glycero-3-phosphocholine (DOPC) and 1,2-dioleoyl-*sn*-glycero-3-phospho-(1′-*rac*-glycerol) (DOPG) were from Avanti Polar Lipids as lyophilised solids. The fluorescent lipid NBD-DHPE (*N*-(7-nitrobenz-2-oxa-1,3-diazol-4-yl)-1,2-dihexadecanoyl-*sn*-glycero-3-phosphoethanolamine) were from ThermoFisher Scientific. The solid salts 4-(2-hydroxyethyl)piperazine-1-ethanesulfonic acid (HEPES, ≥99.5%) and sodium chloride were from Sigma Aldrich/Merck.

### 2.2 CuInS_2_/ZnS synthesis

A solvothermal method was employed to synthesize CuInS_2_/ZnS QDs, utilizing thiolate ligands.^24,27^ Precise control over the optical properties of the QDs was achieved by manipulating the CuInS_2_ QD seeding time and through the growth of a ZnS shell *via* a hot injection process. Typically, 0.25 mol of copper iodide and 0.25 mol of indium acetate were added to octadecene (ODE) and a molar excess of thiolate ligand. The mixture was degassed and stirred under a protective atmosphere to prevent the formation of oxygen–sulfur anti-side defects, then the temperature was raised to 120 °C to dissolve the powder metal precursors. Once dissolved, the temperature was increased to 230 °C to begin nucleation then was lowered to 200 °C for the growth phase. After a predetermined seeding time, the core growth was arrested by rapidly decreasing the temperature. Subsequently, for growth of a ZnS shell, 0.5 mol of zinc stearate was added to 2 ml ODE and 2 ml octanethiol and flushed with argon, then heated to 120 °C under continuous stirring. The ZnS precursor was then injected to the CuInS_2_ core solution and refluxed at 200 °C for 1 hour, at which point the reaction was quenched by rapidly dropping the temperature. To clean, this unrefined solution was diluted tenfold in a 10 : 5 : 1 acetone : methanol : chloroform polar mixture to promote QD precipitation. The polar QD mixture was centrifuged at 4000*g*, with the supernatant discarded and the resulting pellet re-suspended in a minimal amount of chloroform. The cleaning process was repeated twice.

### 2.3 Quantum dot-lipid nanocluster formation

Lipids were suspended in chloroform to give a 20 mg ml^−1^ solution, in a glass vial, then the desired ratio of CuInS_2_/ZnS QDs were added to the aliquots, as calculated from the stock's absorbance at 400 nm and the molar extinction coefficient.^27^ Samples were prepared with a range of lipid : QD ratios in the range from 100 : 1 to 2000 : 1 (mol mol^−1^), with the standard ratio being 1500 : 1 (where not otherwise stated) and concentrations of 6.81 μM QDs and 10.2 mM lipids. The lipids used were a mixture of DOPG and DOPC and preliminary experiments found that the optimal ratio was 2 : 1 DOPC : DOPG to maximize QD stabilization in clusters, so this lipid mix was used for all samples reported herein. If required, the fluorescent lipid NBD-PE was added at this stage (at 1% mol mol^−1^ relative to total lipid). The aliquots of lipids dissolved in chloroform were vortexed multiple times to ensure good mixing and the desired volume of CuInS_2_/ZnS QDs in chloroform was injected. To form a dry thin film of lipids and QDs, a dry nitrogen gas flow was used to enhance chloroform evaporation. The vials of mixed lipids and QDs were placed inside a vacuum desiccator to remove any residual traces of chloroform (covered with aluminium foil at room temperature to prevent photo-bleaching).

The dry QD/lipid thin films in glass vials were solubilized with 0.5 ml of an aqueous solution of 4% w/v sodium cholate detergent, 50 mM HEPES, 100 mM NaCl (pH 7.5), then bath sonicated and vortexed to disperse and form lipid bilayer fragments. The bath sonication and vortex cycle was repeated twice. Samples were added to 1.5 ml plastic microcentrifuge tubes containing degassed nonpolar polystyrene absorbent Bio-Beads, which were then placed in a pinwheel rotator to agitate the mixture during incubation. Four incubation cycles with increasing quantities of Bio-Beads (100 mg, 200 mg, 400 mg, 400 mg for 45 min, 45 min, 45 min, then 18 hours, respectively) were used to gradually remove the detergent and promote the self-assembly of lipids and QDs. The QD–lipid nanocomposites formed were either characterized immediately or stored in the dark at 4 °C.

### 2.4 Electron microscopy

QD/lipid samples were ultrasonically dispersed for ∼5 min and a couple of drops were drop cast onto a Cu grid coated in holey amorphous carbon film with a thin film of graphene oxide (GO). The GO covers the amorphous carbon film and flakes of GO lie across the holes giving an ultra thin film ideal for imaging quantum dots. The grids were allowed to dry in air before imaging. Electron microscopy analysis was carried out using the FEI Titan Themis Cubed instrument, operated at 300 kV. The Titan was fitted with a monochromator which allowed the screen current to be reduced to 2 nA to reduce beam damage and contamination build up. The bright field images were collected on the Oneview 16 Megapixel CMOS digital camera. STEM images were collected using a probe current of 10 pA, a dwell time of 20 microseconds and total scan time of ∼10 min.

### 2.5 Absorption (UV-Vis) spectroscopy

Absorption measurements were performed using a 10 mm pathlength quartz cuvette (600 μL volume) for QD characterization in chloroform and for QD–lipid nanocluster characterization in aqueous buffer. Before measurements, the samples were diluted to an absorbance of 0.1 at 400 nm to avoid inner filter effects using either chloroform (colloidal QDs) or aqueous buffer (50 mM HEPES, 100 mM NaCl, pH 7.5, no detergent). An Agilent Technologies Cary 5000 UV-Vis-NIR absorption spectrophotometer was used, with the temperature controlled at 20 °C. Scans were completed over a 300–800 nm wavelength range, with baseline correction against the appropriate solvent (chloroform or aqueous buffer).

### 2.6 Ensemble fluorescence spectroscopy

Steady-state fluorescence spectroscopy was performed by transferring the aforementioned samples to an Edinburgh Instruments FLS980 fluorescence spectrophotometer equipped with dual excitation monochromators and dual emission monochromators, immediately after absorption spectroscopy measurements. Samples were characterized at 20 °C using a thermoelectrically-cooled cuvette holder (Quantum Northwest TC 1 Temperature Controller). Excitation was achieved using a 450 W Xenon arc lamp and detection using a red-sensitive photomultiplier tube module (Hamamatsu R928 PMT). Emission scans were completed on the CuInS_2_/ZnS QDs using a 500 nm excitation wavelength and collecting between 540–800 nm using a 4 nm bandwidth excitation slit and a 2 nm emission slit. The bandwidth of the excitation and emission slits were increased to 8 nm and 4 nm respectively for QD–lipid nanocluster emission scans. Data acquisition parameters were 0.5 nm steps, integrating 0.1 s per step with three scans averaged to reduce noise.

Time-resolved fluorescence spectroscopy was performed using the same FLS980 instrument. Excitation was achieved using a 473 nm pulsed diode laser, with the pulse repetition rate typically set at 1 kHz (1 ms pulse interval). A built-in neutral density (ND) filter wheel was applied to the pulsed laser, allowing excitation power to be set as desired. Finally, a high-speed red-sensitive photomultiplier tube module (Hamamatsu H10720-20 PMT) was used for detection, centered at a wavelength of 700 nm (unless performing TRES scans where the emission wavelength was scanned across a range, as specified in the text). The settings were optimized so that as low laser power as possible was used to minimize the possibility of photo-bleaching and excitation annihilation effects, whilst still achieving a reasonable signal-to-noise for the fluorescence decay curve.

### 2.7 Epifluorescence microscopy and fluorescence lifetime imaging microscopy

The substrates used for characterizing samples with fluorescence microscopy techniques were 50 × 25 mm glass coverslips (# 1.5 thickness), made hydrophilic by a 30-minute incubation in a “piranha solution” comprised of 3 : 1 mixture of sulfuric acid to hydrogen peroxide, then rinsed with ultrapure water. Before use, cleaned glass coverslips were removed using clean tweezers and dried with a nitrogen gun. For imaging samples under aqueous buffers, a hydrophobic adhesive “imaging spacer” (0.12 mm depth, 9 mm diameter, Grace Bio-Labs) was attached to the dry glass substrate to confine a droplet of buffer, for an open-sample setup, allowing buffer exchanges by pipetting. To ensure effective attachment of the lipid-stabilized QD nanoclusters to the coverslip, positively charged poly-l-lysine (PLL) (Sigma, MW 30 000–70 000) was used to enhance electrostatic interaction between the negatively charged DOPG lipid and the substrate. 100 μl of 0.1% w/v PLL was incubated with the clean glass for 30 min, washed with ten changes of ultrapure water to remove excess PLL, then dried on a hot plate at 40 °C. Meanwhile, lipid-stabilized QD nanoclusters were diluted as required (×1, ×10, ×100 or ×250 diluted from an initial concentration of 5 mg ml^−1^ lipid) using 10 mM HEPES, 20 mM NaCl (pH 7.5). A droplet of the diluted QD–lipid nanoclusters was incubated with the PLL-coated glass coverslip for 15 min. Samples were then washed ten times with 10 mM HEPES, 20 mM NaCl (pH 7.5) and then exchanged into a 50 mM HEPES, 100 mM NaCl (pH 7.5) with three washes of buffer to maintain consistency between microscopy and spectroscopy conditions. For epifluorescence imaging of dried samples, nanoclusters were deposited on PLL-treated coverslips using the same protocol, but after incubation and washing, samples were allowed to air-dry prior to imaging.

Epifluorescence microscopy was performed with a Nikon E600 microscope equipped with a Andor Zyla 4.2 sCMOS detector. Appropriate filter cubes were used to provide selective excitation and emission range for the material of interest: either the CuInS_2_/ZnS QDs (excitation 540–580 nm, emission 600–660 nm) or the NBD–lipid probe (excitation 465–495 nm, emission 515–555 nm). The exposure time, binning and ND filter settings were optimized for each sample.

Fluorescence Lifetime Imaging Microscopy (FLIM) was performed on a PicoQuant MicroTime 200 time-resolved confocal fluorescence microscope (all hardware mentioned below: from PicoQuant). Samples on glass coverslips were placed onto an Olympus IX73 inverted optical microscope, equipped with a ×60 water objective (UPlanSApo, N.A. 1.2×). The excitation sources were 485 nm and 561 nm pulsed lasers driven in Pulsed Interleaved Excitation mode by a PDL 828 Sepia II burst generator module, with repetition rates of 20 MHz and 1 MHz, respectively. The laser power was kept at a low level to prevent photo-damage (30 AU for imaging QDs; 500 AU for NBD). The pulse widths for the 485 nm and 561 nm lasers were 90 ps and 70 ps, respectively. The fluorescence emission from samples was collected through the same objective lens and then transmitted through an appropriate dichroic mirror towards a detection arm of the optical path. The fluorescence from NBD was collected through a 520/35 bandpass filter by a hybrid PMT detector and fluorescence from CuInS_2_/ZnS QDs was collected through a 690/70 bandpass filter by a single-photon avalanche diode detector. Timing electronics were a TimeHarp 260 module. Images were collected using a “FLIMBee” mirror-based galvanometer scanner, typically with 256 × 256 pixels at the desired magnification (often 50 × 50 μm to find regions of interest and 5 × 5 μm to observe individual QD–lipid nanoclusters). A series of image “frames” were collected sequentially and accumulated to generate a high quality final image (100 frames, 25 μs dwell time per pixel, 164 s total acquisition time per image). Analysis of fluorescence decay curves was performed using SymPhoTime software (PicoQuant) to fit a bi-exponential decay function that was re-convoluted with measured IRFs (good fits were achieved with *χ*^2^ ∼ 1). The amplitude-weighted mean lifetime was calculated, to reflect the quenching of the energy donor due to FRET.

## 3 Results and discussion

### 3.1 Developing a method to assemble and stabilize quantum dots as clusters within aqueous solutions

Our concept was that it should be possible for QDs with hydrophobic exterior surfaces to be stabilized in water by surrounding them with lipids. Lipids were chosen because they are small amphiphilic molecules that are known to self-assemble and are of a similar size (2 nm molecular length) to a single QD (2–10 nm particle size, typically).^69^ It seemed likely that the hydrophobic tailgroup of lipids would associate with the apolar ligands of QDs and that the hydrophilic headgroup of lipids would face towards the aqueous solvent, but it was uncertain how they would organize or the size of structures that would form. We assumed that if assembly process was not controlled then the particles may quickly form massive aggregates and simply sediment out of solution. In an effort to control the process, we took inspiration from typical procedures used to prepare lipid vesicles,^70,71^ where lipids are dispersed in an organic solvent, dried to a thin film on a solid surface, then finally rehydrated into an aqueous solution, and adapted this method to include QDs. Other studies had found success with similar procedures^64^ and we hoped to optimize this for our specific QDs.

**Fig. 1:**
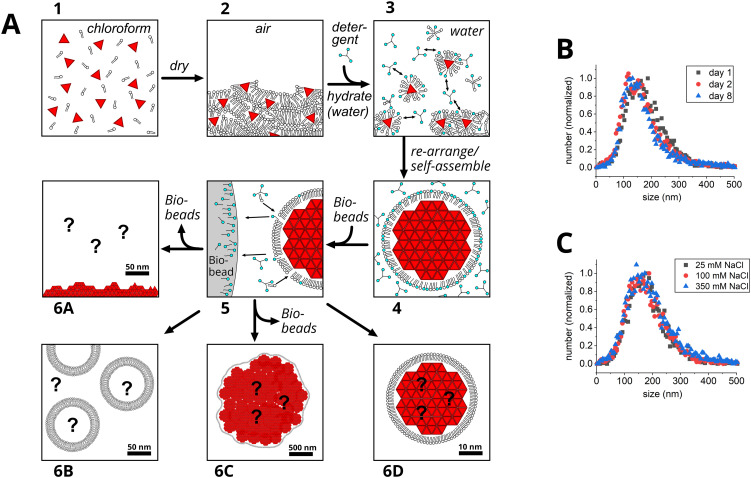
Initial consideration of the possible sizes of the assemblies that may form. (A) Cartoon illustrating the process of QD cluster formation, portraying potential structures at each stage of self-assembly. QDs are represented as red triangles, lipid molecules as a headgroup with two tails in black, detergent as a headgroup with one tail in cyan. Panel (1) shows a colloidal distribution of QDs and lipids dispersed in chloroform. Panel (2) shows a dried thin film of QDs and lipids. Panel (3) shows the expected formation of small QD clusters upon re-hydration of the film with 4% detergent. Panel (4) shows a continuation of the QD stabilization *via* the formation of mixed lipid/detergent micelles, with excess detergent present in the buffer. Panel (5) shows the removal of the detergent using adsorbent Bio-Beads. Panel (6) illustrates the possible end states for the QDs following removal of the detergent, where the scale bars suggest our expectation of the sizes of structures that may form. The four options shown are: precipitating out of solution due to a lack of stabilization (Panel 6A), the formation of empty lipid vesicles where QDs have not been stabilized by the lipid (Panel 6B), larger QD aggregates stabilized by lipids (Panel 6C), and smaller clusters of QDs stabilized by a monolayer of lipids (Panel 6D). (B) NTA data comparing the size of QD–lipid assemblies at various timepoints, in a buffer of 50 mM HEPES, 100 mM NaCl, pH 7.5. (C) NTA data comparing the size of QD–lipid assemblies in buffers containing various salt concentrations, on the day of preparation. Further details of the NTA measurements are provided in Fig. S2.

Fig. 1A details the new process that we developed for the formation of QD–lipid clusters, showing the potential structures that may form at each stage of the process. The process is described below, and then our characterization of the samples produced at the end stage is presented later in the text. The starting materials were lipids that had been purchased in a solid form and simply dissolved in chloroform and QDs that were chemically synthesized in-house and purified in chloroform (see Experimental section). In the first step of the assembly procedure, colloidal QDs and lipids were mixed together in chloroform as a suitable solvent that fully disperses both components (Fig. 1A, state 1). Subsequently, a dried thin film of QDs and lipids should be obtained by exposing the mixture to a constant flow of dry nitrogen gas. We imagined that there would be a multi-layer film of lipids interspersed with individual or clustered QDs (Fig. 1A, state 2). The next stage was to attempt to take QDs and lipids into a liquid phase again, but this time to a water-based environment, by adding an aqueous solution of salt and detergent and vigorously agitating the tube (vortexing, sonication). This is known to work well for lipids but our preliminary work found that this was more challenging for QDs. Once the mixing procedure and choice and concentration of detergent was optimized we were able to obtain a cloudy solution that we assumed must contain large (*e.g.*, microscale) aggregates (Fig. 1A, state 3–4). We anticipated that the surfactant would stabilize the QDs in some combination with the lipid. An excess of detergent and lipid was required, therefore, mixed lipid/detergent micelles and QD/lipid/detergent assemblies would be in some sort of dynamic equilibrium. The detergent was then removed using polystyrene adsorbent beads (“Bio-Beads”) that are thought to selectively remove the small detergent molecules in preference to the larger lipid molecules (Fig. 1A, state 5). We hoped that following detergent removal we would achieve a stable end-state for the QDs and considered a few possibilities, shown in state 6A–6D of Fig. 1A: (a) precipitation out of solution due to a lack of stabilization, (b) formation of empty lipid vesicles, (c) formation of very large QD aggregates stabilized by lipids, and (d) formation of relatively small clusters of QDs stabilized by a monolayer of lipids. We focussed our characterization methods on this end state of the QD–lipid assemblies and did not attempt to assess the intermediate stages in detail. Preliminary investigations determined a suitable ratio of lipid-to-QD to generate stable assemblies (1500 : 1 mol mol^−1^) and measurements of the absorbance and fluorescence of control samples supported the assembly process described above (see Fig. S1 in the SI).

Particle sizing with dynamic light scattering was attempted but the data was unreliable due to complicating fluorescence signals from the QDs. Instead, the Nanoparticle Tracking Analysis (NTA) technique was used for an initial assessment of the size of QD–lipid assemblies formed in aqueous solution (*i.e.*, after detergent removal in state 5 in Fig. 1A). NTA of particles studied soon after their assembly revealed a moderately broad distribution of sizes with a peak at 160 ± 70 nm (see Fig. 1B). The size of clusters decreased slightly after their storage for extended periods of time, reaching 130 ± 60 nm after 7 additional days (see Fig. 1B). The presence of salt did not significantly change the particle size or stability, considering a range from 25 to 350 mM NaCl, whether assessing newly-assembly particles or on day 8 (see Fig. 1C and Fig. S2). Zeta potential measurements also showed that the particles were stable on the day they were formed (approx. −40 mV), with some reduction after 7 additional days (see Fig. S3). All subsequent analysis of QD–lipid assemblies was performed on samples within 24 hours of production and using a buffer of intermediate ionic strength (50 mM HEPES, 100 mM NaCl, pH 7.5). Overall, this particle sizing analysis suggested that the QD–lipid assemblies were roughly 100–200 nm and relatively stable. However, the accuracy of the mean and distribution was uncertain because NTA is known to be biased towards detecting larger particles. For particles with similar refractive index, the scattered-light signal detected in NTA increases approximately in proportion to the sixth-power of particle diameter (signal ∝ d^6^). For example, a 200 nm particle is expected to produce roughly 4096 times the signal of a 50 nm particle with NTA. Practically, this leads to a ∼20 nm detection limit under typical conditions.^72,73^ Consequently, in a polydisperse sample, this leads to preferential visibility of larger particles, which in turn overestimates the average size.^72,73^ If there was heterogeneity in the composition of particles assembled in the current work, such as an uneven distribution of lipid and QDs across the population of particles, then this would not be well represented by NTA data. Balancing these merits and demerits of the technique: the NTA analysis allowed us to rule out major precipitation of material (state 6A in Fig. 1A) and microscale aggregates (state 6C in Fig. 1A), but we could not distinguish between lipid-only and QD–lipid particles (states 6B and 6D in Fig. 1A). The detailed analysis in later sections clarifies this.

### 3.2 Structure and optical properties of clustered quantum dots *versus* individual quantum dots

**Fig. 2:**
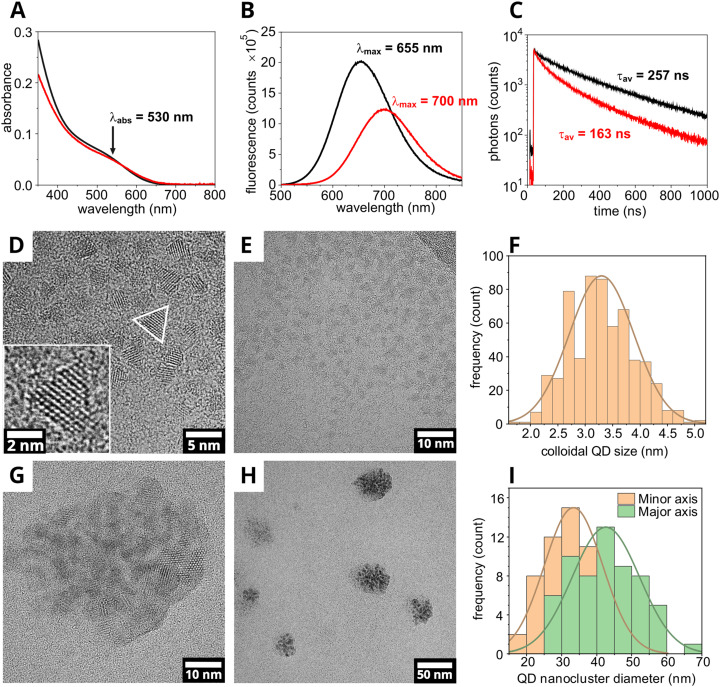
Spectra and transmission electron microscopy (TEM) images showing alteration of optical properties and structure for colloidal CuInS_2_/ZnS QDs and clusters of CuInS_2_/ZnS QD. (A) Absorption spectra, (B) steady-state fluorescence emission spectra (*λ*_ex_ = 475 nm), and (C) semi-logarithmic plot of the fluorescence decay curves of colloidal CuInS_2_/ZnS QDs in chloroform (black) and clusters of CuInS_2_/ZnS QDs in aqueous solution (red). For fluorescence decay curves, there was laser excitation at *λ*_ex_ = 473 nm and photon collection centred either at *λ*_em_ = 655 nm (colloidal QD) or 700 nm (clustered QD). Curves were fit to a multi-exponential decay function and the mean amplitude-weighted lifetime was calculated. TEM images of colloidal CuInS_2_/ZnS QDs with (D) 5 nm scale, (inset) a magnified portion of an image isolating an individual QD with 2 nm scale, and (E) a 10 nm scale. (F) Histogram of the colloidal CuInS_2_/ZnS QD particle size, defined as the longest corner-to-side distance of the triangular projection. A Gaussian fit of the distribution is shown. TEM images of CuInS_2_/ZnS QD nanoclusters with (G) a 10 nm scale and (H) a 50 nm scale. (I) Histogram of the CuInS_2_/ZnS QD nanocluster diameter, taken along the minor (orange) and major (green) axes of the elliptical aggregates. A Gaussian fit of each distribution is shown. Further details of the TEM and STEM analyses are provided in Fig. S5–S10.

First, we will describe the properties of individual “colloidal” QDs and then compare these to the properties of the assemblies formed by our new procedure. The first prominent absorption feature, sometimes called the “first excitation peak” by QD researchers, was observed as a side-peak at approx. 530 nm for colloidal QDs (Fig. 2A, black line), with a fluorescence emission maximum at 655 nm (Fig. 2B, black line). Time-resolved fluorescence measurements taken on the colloidal CuInS_2_/ZnS QDs show a 257 ns average lifetime (Fig. 2C, black line). To quantify the size of QDs (and later QD clusters), electron microscopy (EM) imaging was performed. High-resolution EM images revealed that the CuInS_2_/ZnS QDs, as synthesized, had the expected tetrahedral structure represented by a triangular shape in the 2-D projection images (white triangle, Fig. 2D). The lattice fringes were clear in many particles (inset, Fig. 2D) and the spacing was measured as 0.326 ± 0.006 nm (*n* = 10, mean ± S.D.), consistent with previous measurements of the (112) plane of chalcopyrite CuInS_2_ cores, for example, 0.32 nm measured by Booth and co-authors for CuInS_2_ QDs.^27^ Several EM images were analyzed to determine the size distribution of the colloidal form of the nanoparticle (Fig. 2E and F), resulting in an average particle size of 3.30 ± 0.59 nm (*n* = 741, mean ± S.D.), with a size dispersion of 18% (standard deviation as a percentage of the mean) determined through semi-automated analysis of TEM (Transmission Electron Microscopy) and STEM (Scanning Transmission Electron Microscopy) images (see Fig. S5–S8 for further images and details of the analysis protocol). The colloidal QDs appeared to associate with the edges of the TEM grid due to the effect of drying the particles onto the carbon grids required for TEM. Images acquired in scanning-TEM mode provided higher contrast, albeit at a lower spatial resolution, and correlated with the findings of standard TEM images (see Fig. S7).

Having established the structure and optical properties of the individual QDs, our lipid/QD co-assembly procedure was performed and the characterization process was repeated. The first absorption peak of the lipid-stabilized QD clusters was at a similar position to the isolated colloidal QDs (Fig. 2A, red line). Subtle differences in the shape of absorption spectra could be due to scattering effects in the QD–lipid samples (fluorescence excitation spectra show similar shapes, see Fig. S4). A significant red-shift of 45 nm in the fluorescence emission maxima of the clustered QDs to 700 nm was observed, attributed to QD agglomeration (Fig. 2B, red line). This sort of fluorescence red-shift has been observed in a few previous studies^41–43^ and could be caused by energy transfer among QDs, but the size of the structures is not revealed by the spectroscopy data. Time-resolved fluorescence measurements reveal a faster decay rate for clustered QDs (Fig. 2C, red line) *versus* isolated QDs, resulting in an average fluorescence lifetime of 163 ns, compared to 257 ns for colloidal QDs. TEM images revealed that the structures formed by the self-assembly procedure were relatively small QD ‘nanoclusters’ ranging in size from 20 to 60 nm (Fig. 2G and H and Fig. S9). Measurements of many particles revealed that the clusters were elliptical, the diameter of minor axis and the major axis were 33.4 ± 8.3 nm and 42.6 ± 9.6 nm (*n* = 60, mean ± S.D.), respectively, which gives a modest size dispersion of 23–24% (Fig. 2I). These nanoclusters displayed a notably higher electron density compared to colloidal QDs, presenting darker structures, indicative of a three-dimensional aggregate of overlapping particles, as opposed to the two-dimensional arrangement sometimes seen due to the effect of drying colloidal QDs onto the TEM grids. Consistent with the TEM data for colloidal QDs, the structure of individual QDs within each nanocluster was clearly discernible, thus affirming the presence of QDs. Again, STEM mode provided higher contrast images that confirmed the structure of these QD aggregates (see Fig. S10).

For the QD clusters, the size of 30–50 nm measured by TEM is significantly smaller than size suggested by the NTA particle sizing data (100–200 nm). This difference is likely to reflect the fact that TEM primarily probes the dry nanoparticle core, whereas the NTA particle sizing measures the hydrodynamic size of lipid-associated structures in solution, including hydration and potential higher-order assemblies. TEM will observe the core clusters of QDs but is unlikely to detect a loose lipid corona surrounding them. There could be significant amounts of lipid-only vesicles (formed due to an excess of lipids over QDs) that are detected by NTA but not observed by TEM. These possibilities are assessed in section 3.3.

It is important to note that spectra of a mixture of QDs and lipids in chloroform (a solvent expected to fully disperse each component) are identical to spectra of QDs alone in chloroform (Fig. S1). The spectral shift occurred only after the assembly procedure involving transfer of QDs to an aqueous solvent. This observation suggests that assembly of QDs into clusters is primarily driven by the change in the polarity of the solvent, where water is an unfavourable environment for QDs with this hydrophobic surface chemistry (octanethiol ligands), which leads individual nanoparticles to associate. Dried films of QDs could be solubilized by a detergent solution without lipids but, unsurprisingly, QDs were unstable and precipitated out of solution if the detergent was removed (Fig. S1). Conversely, detergents were required to properly dissolve the dried QD–lipid film and attempts to dissolve the film with an aqueous solution without detergent resulted in a turbid solution where the lipid and nanoparticles were presumed to be poorly dispersed (Fig. S1). This underscores the essential roles of both detergents and lipids in the self-assembly process. This suggests that our procedure utilizing lipids was successful in stabilizing QDs in small clusters.

### 3.3 Colocalization of lipids and quantum dots within nanoclusters

**Fig. 3:**
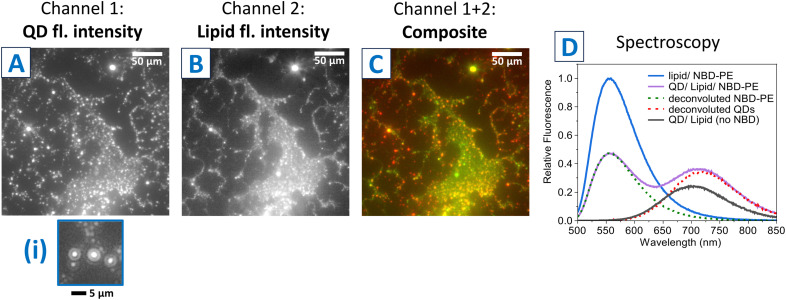
Fluorescence microscopy and spectroscopy analysis of the colocalization of lipids and quantum dots. (A) An example low-magnification epifluorescence image of QD/lipid/NBD-PE nanoclusters dried onto a glass coverslip. This image channel represents the fluorescence from QDs (excitation at 540–580 nm, emission at 600–660 nm). (i) Magnification of a region within (A) to show single particles. (B) Epifluorescence image of the same field of view as in (A), except in the image channel representing the fluorescence from the lipid probe NBD-PE (excitation at 465–495 nm, emission at 515–555 nm). (C) Composite image of (A) and (B), displaying the signal from QDs in red and the signal from lipids (NBD) in green. (D) Fluorescence emission spectra of either lipid-only vesicles (blue), QD/lipid nanoclusters (black) or QD/lipid/NBD-PE nanoclusters (purple). These spectra were acquired with excitation at 475 nm, which is selective for NBD. The dashed lines (green, red) represent a deconvolution of the two peaks found in the data from the QD/lipid/NBD-PE nanoclusters sample.

Whilst QDs were readily observable in TEM images, as they are highly electron-dense, it was not possible to locate the lipids by this technique (the much lower electron density of lipids typically provides little contrast in TEM imaging). Therefore, the next step was to visualize the location of the lipids compared to the QD clusters. To do this, a small quantity of fluorescently-labelled lipids were included: 1% mol mol^−1^ of NBD-PE relative to the standard lipids. The fluorescence of this lipid probe could be distinguished from the fluorescence of the QDs by using an epifluorescence microscope with suitable filter sets. The QD/lipid/NBD-PE nanoclusters were deposited onto glass coverslips, rinsed with water, and then dried. Fluorescence images showed that QDs spread across a surface over large regions of hundreds of microns (Fig. 3A) and discrete bright spots could be observed, presumed to be individual nanoclusters. The QD fluorescence appeared to be diffraction-limited (Fig. 3A, (i)), as expected for (nanocluster) particles of approx. 50 nm in diameter (as determined from TEM) considering this simple microscope had a spatial resolution of approx. 1 μm. In contrast, the fluorescence signal from the lipid seemed to spread over a larger area and often appeared to connect between the regions of QD signal (Fig. 3B). These connections could have been artifacts of drying the particles onto the glass substrate and may not have existed when the QD–lipid clusters were suspended in water (clarified later in this study). Composite images confirmed that the lipid fluorescence was co-located with the QD fluorescence (Fig. 3C). Next, spectroscopy measurements were performed to assess how close the lipid-probe was to the QDs *via* a simple FRET measurement. The NBD probe was chosen because it should act as a FRET donor to the QDs, which should act as a FRET acceptor. Therefore, if the lipids were present in close proximity to the QDs then the fluorescence from NBD probe should be quenched. Steady-state fluorescence spectra of NBD-PE either in the presence or absence of the QDs nanoclusters showed a “donor quenching” of approx. 52% (Fig. 3D, blue *vs.* green line), leading to a mean separation distance of ∼5 nm between NBD-labelled lipids and QDs (calculated from simple FRET relationships, where 5 nm is the estimated Förster radius).

The QD fluorescence peak also appeared to be somewhat higher due to the presence of NBD (Fig. 3D, red *vs.* black line), and such “acceptor enhancement” is also good evidence of FRET. Overall, these analyses imply that a significant quantity of lipid was within a few nanometres of a QD.

**Fig. 4:**
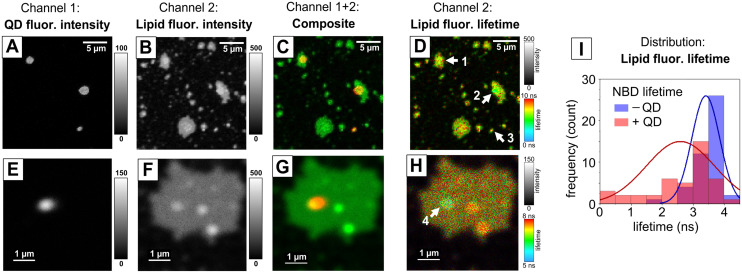
Fluorescence lifetime imaging microscopy (FLIM) analysis of lipid and QD colocalization, performed with hydrated samples (under aqueous buffer). Some panels display solely the fluorescence intensity (grey-scale) and some panels display the fluorescence lifetime (colour-scale). (A) An example image of QD/lipid/NBD-PE nanoclusters at medium magnification, in a channel representing fluorescence intensity only from QDs (561 nm laser excitation, 655–725 nm emission collection range). (B) Image of the same field-of-view as in (A), except displaying the channel representing fluorescence intensity from NBD-PE (485 nm laser excitation, 502–537 nm emission collection range). (C) Composite image of (A) and (B), displaying the signal from QDs in red and the signal from NBD-PE in green. (D) Fluorescence lifetime image of NBD-PE, *i.e.*, the time-resolved data that matches (B). Arrows denote positions-of-interest within the image where the QD and lipid fluorescence appears to be co-located. (E)–(H) QD/lipid/NBD-PE nanoclusters at higher magnification, displaying the same channels as for (A)–(D). (I) Frequency distribution histogram plot of the fluorescence lifetime of the NBD-PE in the presence (red) or absence (blue) of QDs. This plot was generated by careful analysis of many particles, as described in the SI (see Fig. S11 and S12).

To confirm the colocalization of lipids, we used an alternative fluorescence microscope that could acquire images of higher spatial resolution and quantify the fluorescence lifetimes (FLIM). The QD/lipid/NBD-PE nanoclusters were deposited onto glass coverslips, rinsed, and imaged by FLIM. This instrument allowed us to observe the nanoclusters without drying them onto a surface, which was important to avoid the possible drying artifacts observed in epifluorescence images that may have altered the structures of the QD–lipid assemblies. In these higher quality, higher-resolution images it was clear that there were fewer spots of QD fluorescence (Fig. 4A) than lipid fluorescence (Fig. 4B, same field of view). There were several patches of lipid in regions where there was no QD fluorescence (Fig. 4C), suggesting that “empty” lipid vesicles had assembled, attributed to the relatively high lipid : QD molar ratio that was chosen to promote the complete encapsulation of QDs. This is consistent with the observation of larger sized particles by NTA (Fig. 1B) because lipid vesicles are often 100–200 nm in diameter and readily rupture onto glass substrates and fuse together to generate microscale patches of lipid bilayers.^74^ In future studies, it may be possible to separate and remove these lipid-only vesicles *via* differential centrifugation, if desired. It was notable that wherever there was QD fluorescence there was always lipid fluorescence, giving evidence that lipid was required to stabilize the QDs. Higher magnification images make this even clearer where a spot of QD fluorescence (clearly less than 1 μm, probably diffraction-limited) sits on top of a large lipid patch (several microns large) (Fig. 4E–G).

The fluorescence lifetime signal in FLIM images can provide evidence of FRET occurring in specific locations. The presence of QDs appeared to cause a subtle lowering of the fluorescence lifetime of the NBD–lipid, apparent for at least some particles, with a shift from green/red pixel-colour for lipid-only regions (6–7 ns) to blue/green pixel colour (5 ns) where there was a QD nanocluster (Fig. 4D, particle #2 is clear). In higher magnification images, some particles showed a significantly reduced lifetime (Fig. 4H, particle #4 is clearly blue). This change in lifetime was quantified by manually selecting regions of lipid fluorescence where QDs were co-located and comparing to regions where there were no QDs (see explanations of the analysis approach, Fig. S11 and S12 in the SI). A histogram of NBD lifetimes shows a wide distribution but there is clearly a shift to overall lower lifetimes in the presence of the QDs (Fig. 4I red *vs.* blue distribution). This reduction in lifetime of NBD is strong evidence of FRET from lipid-linked NBD to QDs, confirming the close proximity of lipids to QDs. The wide range of lifetimes correlates with the idea that some lipid vesicles had formed without any QD associations (long lifetime, representing NBD far from QDs) and some lipids had associated very closely to QDs (explaining the short lifetimes of 0–1 ns). Indeed, if there was a lipid monolayer surrounding each nanocluster of QDs then each NBD moiety (attached to the headgroup of the lipid) would be within about 2 nm of the QD shell (state 6D in Fig. 1A). A more detailed discussion of the NBD lifetime changes due to QDs and a plot of the intensity *versus* lifetime of single-particles is provided in the SI (see Fig. S13 and associated text). Overall, these fluorescence microscopy studies prove that the stabilisation of QD nanoclusters required close associations with lipids.

### 3.4 Importance of lipid self assembly for stablizing quantum dots

**Fig. 5:**
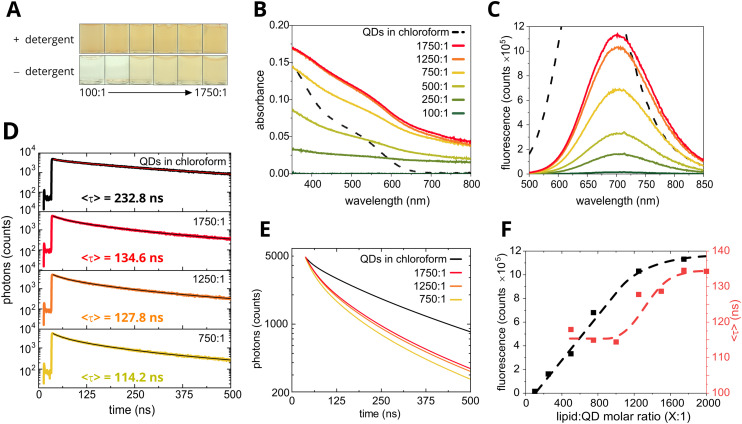
Investigating the effect of lipid-to-QD ratio on the optical properties of QD–lipid nanoclusters. (A) Photograph of glass vials containing samples undergoing the procedure for the self-assembly of QD–lipid nanoclusters, either before (top) or after (bottom) detergent removal by adding Bio-Beads. Images from left to right show an increasing ratio of lipid : QD (mol mol^−1^) of 100 : 1, 250 : 1, 500 : 1, 750 : 1, 1250 : 1 and 1750 : 1. (B) Absorbance spectra of QD nanoclusters in aqueous buffer, at a range of lipid : QD ratios from 100 : 1 (dark green) to 1750 : 1 (dark red) with a fixed QD concentration. A sample of colloidal QDs dissolved in chloroform is shown for comparison (dashed black). (C) Accompanying steady-state fluorescence emission spectra (*λ*_ex_ = 475 nm) of the same QD nanocluster samples from (A), with matching colours. (D) Fluorescence decay curves of a series of samples comparing colloidal QDs in chloroform (black) to QD–lipid nanoclusters with varying lipid-to-QD ratio (same colouring as (A)). Laser excitation at *λ*_ex_ = 473 nm and photon collection centred either at *λ*_em_ = 655 nm (colloidal QD) or 700 nm (clustered QD) was used. The mean amplitude-weighted lifetime is displayed. The fitting of all fluorescence decay curves to multi-exponential decay functions is shown in Fig. S14. (E) Graph showing the fits of the fluorescence decay curves from panel D, overlaid to show differences between the samples. (F) Graph comparing how the steady-state fluorescence intensity (black) and the mean fluorescence lifetime (red) of QD–lipid nanoclusters varies with the lipid-to-QD ratio. For all samples, 2 : 1 mixture of DOPC : DOPG lipids was used because this appeared to be the optimal balance of charged and neutral lipids (see Experimental section).

To assess the influence of lipid–QD interactions on the formation of QD clusters, we systematically varied the lipid-to-QD molar ratio during the assembly process. A series of samples were prepared with a fixed QD concentration while adjusting the lipid concentration, leading to a lipid-to-QD ratio range from 100 : 1 to 1750 : 1 (mol mol^−1^). Fig. 5A shows photographs of the glass vials containing the samples, providing visual evidence of the presence of QDs and how its concentration varies with increasing lipid ratio. All samples had similar coloration in the presence of the stabilizing detergent, irrespective of the different amounts of lipids present in solution (Fig. 5A, top row). However, after detergent had been removed during the protocol to assemble QD–lipid nanoclusters, it was apparent that the concentration of QDs was reduced in all samples, from the reduced coloration, but the degree of loss varied greatly between samples (Fig. 5A, bottom row). In the samples with lowest lipid : QD ratio (100 : 1 and 250 : 1) the vial appeared almost colourless, with the coloration intensifying as the amount of lipid was increased, eventually resembling the mixture with detergent for the highest lipid : QD ratios (1250 : 1 and 1750 : 1). Absorption measurements (Fig. 5B) unveiled crucial insights into the amounts of lipids required to stabilize QD clusters. A low absorption signal was observed at a 100 : 1 lipid-to-QD ratio (dark green line), indicating that little material was present and suggesting lower QD stability at lower lipid concentrations, in agreement with the photographic evidence. The 250 : 1 and 500 : 1 lipid-to-QD ratios (forest green and lime green lines) exhibited greater absorption but lacked a prominent first excitation peak, implying that the apparent absorbance was actually due to light scattering. This smooth curve of increasing absorbance at low wavelengths was notably different compared to control measurements of colloidal QDs in chloroform where there is a prominent inflection at 520 nm (black dashed line). A marked increase in absorbance was observed at the 750 : 1 lipid-to-QD concentration (yellow line), followed by an apparent maximal absorbance between the 1250 : 1 and 1750 : 1 lipid-to-QD ratios (orange and red lines), indicating a maximum QD incorporation into the lipid assembly. These observations led to the conclusion that QD stabilization requires a sufficient quantity of lipids; QD form large, unstable aggregates that precipitate out of the solution when insufficient lipid is available, resulting in low absorbance at low lipid-to-QD ratios.

Steady-state fluorescence measurements (Fig. 5C) revealed an increase in sample fluorescence intensity with increasing lipid content, with a smaller intensity increase between the 1250 : 1 and 1750 : 1 lipid-to-QD ratio samples, consistent with absorption measurements. This consistency indicated that the fluorescence spectra change was mainly due to variations in the QD concentration, rather than alterations in the energy transfer pathways or significant structural changes. Time-correlated single-photon counting measurements of the fluorescence decay (Fig. 5D) corroborated the steady-state fluorescence findings. These measurements revealed a significant reduction in the fluorescence lifetime for all samples of QD nanoclusters as compared to colloidal QD in chloroform (Fig. 5D and E, colored lines *vs.* black line). A subtle reduction in fluorescence lifetime was evident as the lipid concentration was decreased, with the 1750 : 1 sample exhibiting the least quenching (longest lifetime) of all the QD–lipid nanocluster samples.

Plotting the fluorescence emission intensity against the lipid-to-QD molar ratio (Fig. 5F, black line) revealed a roughly linear increase in fluorescence emission intensity with increasing lipid-to-QD ratio up to 1250 : 1, and then approaching a plateau between a lipid-to-QD ratio of 1250 : 1 and 1750 : 1 at which point the maximum possible amount of QD stabilization was presumably reached. The plateauing of the fluorescence intensity echoes the conclusions drawn from absorption measurements in Fig. 5B. Interestingly, a plot of fluorescence lifetime against the lipid-to-QD molar ratio displayed two distinct phases (Fig. 5F, red line): a low-lifetime level at ∼115 ns and a high-lifetime level at ∼130 ns with an intermediate phase between them. An explanation for the combination of all of the spectroscopy data on these samples is as follows: (i) at the very lowest lipid-to-QD ratio (100 : 1 and 250 : 1) the majority of the QDs had sedimented so absorbance and fluorescence intensity were minimal and a fluorescence lifetime measurement could not be made, (ii) at low and modest lipid-to-QD ratio (500 : 1 to 1000 : 1) there was some limited stabilization of QDs and large aggregates formed resulting in a relatively high level of energy dissipation which caused a very low fluorescence lifetime, (iii) there was a transition where more and more QDs were stabilized which increased the absorbance and fluorescence intensity and also increased the fluorescence lifetime (1250 : 1 and 1500 : 1), (iv) full stabilization was achieved at the highest lipid-to-QD ratios tested and the absorbance, fluorescence intensity and lifetime all appeared to plateau (1750 : 1 and 2000 : 1). An increase in QD lifetime as lipid concentration increased could be attributed to lipid-mediated control of cluster size, suggesting that QD clusters decrease in size with increasing lipid concentration until a stable number of QDs form small clusters when there is an excess of lipid. In these stable, small clusters we surmise that there is a modest amount of downhill energy transfer and dissipation at trap sites which reduces the fluorescence lifetime to lower than colloidal QDs (135 *vs.* 238 ns) but, where the amount of lipid available is limited, the size of the QD clusters increases slightly which allows a larger network for energy transfer that leads to greater energy dissipation which is represented as a slightly lower fluorescence lifetime (114 ns). From 1750 : 1 to 750 : 1 lipid : QD we suggest that there is *a subtle increase* in the cluster size, supported by fluorescence lifetime changes (Fig. 5D–F), although, no size change could be resolved by NTA (Fig. S2), presumably due to the preferential visibility of larger lipid-only vesicles in NTA data as discussed earlier. The *very large increase* to QD cluster sizes at 250 : 1 and 100 : 1 lipid : QD was clear from the absorbance data (Fig. 5B) representing an almost complete loss of material, due to sedimentation of microscale particles where the amount of lipid was insufficient.

### 3.5 Investigating the photophysics of quantum dot nanoclusters

**Fig. 6:**
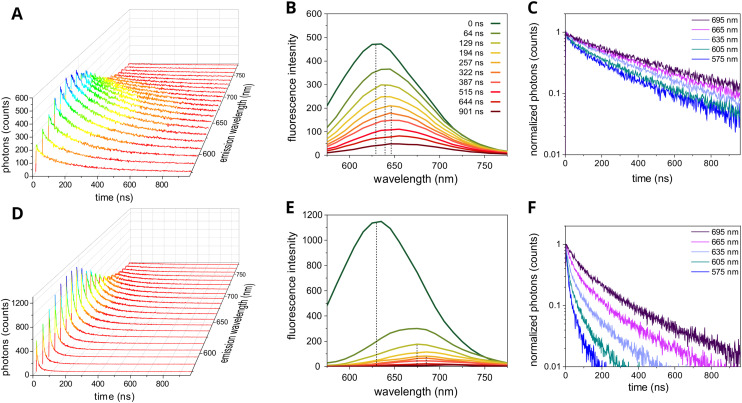
Time-resolved emission spectroscopy (TRES) comparison of the colloidal and clustered forms of CuInS_2_/ZnS QDs. TRES measurements of (A) colloidal QDs in chloroform and (D) QD–lipid nanoclusters in aqueous buffer. Cross-sectional TRES slices taken at ten selected decay times for (B) colloidal QDs and (E) QD–lipid nanoclusters. Black dashed lines represent a guide to the emission peak wavelength at 0 ns, 129 ns, and 257 ns. TRES slices taken at five selected wavelengths for (C) colloidal QDs and (F) QD–lipid nanoclusters. Slices in (C) and (F) were plotted starting from the maximum signal at ∼35 ns to 975 ns and a consistent number of bins was used for each slice (all curves were aligned to start at *t* = 0).

After determining the architecture of QD–lipid nanoclusters, we delved into their ensemble photophysics to investigate the energy transfer pathways. This investigation relied on a combination of fluorescence excitation *versus* emission data and time-resolved emission spectroscopy (TRES) to shed light on these pathways within the nanoclusters. Fluorescence excitation *versus* emission plots solidified the finding of a 50 nm redshift for nanoclustered QDs compared to individualized QDs but, otherwise, produced a straightforward result that the QD emission peak position was independent of the excitation wavelength used, until the excitation wavelength encroached on the emission peak (Fig. S15). TRES measurements were more revealing, allowing us to discern the differences in excited state kinetics between QD nanoclusters and colloidal QDs. Fig. 6A–C and 6D–F depict the results obtained at various emission wavelengths for colloidal QDs in chloroform solution and QD nanoclusters in aqueous solution, respectively.

A 3-D rendering of the TRES scans revealed that the fluorescence decay was significantly more rapid at all collection wavelengths for the QD nanoclusters as compared to the colloidal QDs (Fig. 6D*versus*6A) but, somewhat surprisingly, the peak emission wavelength at time zero was ∼630 nm for both QD arrangements. To separate the time-dependence from the wavelength-dependence the data can be displayed as “slices” at a defined time or wavelength. For the colloidal QD sample, there was a gradual red-shift of the emission peak from ∼630 nm at time zero to ∼645 nm at 257 ns and ∼655 nm at the longest timepoints (dark green to orange to dark red lines, Fig. 6B). This suggests that there was a distribution of QDs that had slightly different energies (due to different sizes) and that, for well-dispersed nanoparticles, the fluorescence from low-wavelength/high-energy QDs was more prominent at shorter timepoints and that the fluorescence from high-wavelength/low-energy QDs tended to occur later. These three peak wavelengths would represent CuInS_2_ core sizes of 2.53 nm (*λ* = 630 nm), 2.61 nm (*λ* = 645 nm) and 2.71 nm (*λ* = 655 nm), if calculating the QD size based on its known relationship with fluorescence emission peak (where “size” is defined as the longest corner-to-side distance of the triangular projection of the QD in TEM images).^27^ For the QD–lipid nanoclusters sample, at time zero the fluorescence emission peak resembled that of dispersed QDs (*λ* = 630 nm), but the red-shift became significantly larger as time increased and the peak moved to ∼675 nm at *t* = 129 ns and to 685 nm at 322 ns (pale green and orange lines, Fig. 6E). These longest wavelengths would represent emission from CuInS_2_ core sizes of 3.00 nm (*λ* = 675 nm) and 3.20 nm (*λ* = 685 nm).^27^ This strongly suggests that there was FRET within the QD clusters from high-to-low-energy QDs and that fluorescence emission from small sub-populations of larger QDs becomes prominent.

The rapid decrease in fluorescence intensity over time, as seen in the cross-sectional data for QD nanoclusters compared to colloidal QDs, may indicate non-radiative energy dissipation (peak heights, Fig. 6B*vs.*6E). This dissipation could result either from phonon scattering following energy transfer or energy transfer to non-luminescent QDs within the cluster.^75–78^ If energy is transferred to QDs while they are in an ‘off’ (non-luminescent) state the fluorescence may be quenched, causing a significant drop in emission intensity and fluorescence lifetime. Supporting this theory, the fluorescence decay curves were relatively shallow for all samples of colloidal QDs (Fig. 6C) and became steeper for QD nanoclusters (Fig. 6F), indicating significant fluorescence quenching. Moreover, the time-resolved measurements reveal that the decay in QD nanoclusters was much more rapid at lower collection wavelengths, aligning with the concept that FRET involves high-energy donor QDs. As the energy is transferred to lower-energy QDs, the fluorescence decay becomes shallower (*i.e.*, slower) at longer collection wavelengths, ultimately resembling that of colloidal QDs at wavelengths above 695 nm.

The fluorescence data presented is in agreement with previous investigations into the energy transfer pathways within thin films of close-packed assemblies of QDs^42–44^ with a faster decay observed in small-blue QDs due to FRET and a slower decay large-red QDs, strong evidence of downhill energy transfer from ‘blue’ to ‘red’ QDs. QD-to-QD FRET within this nanocluster system is believed to occur due to exciton hopping between neighbouring QDs, where energy is transferred to nearby QDs that lie within the Förster radius.^45^ Other authors have observed a different extent of red-shift for the fluorescence emission peak of QDs (when comparing individual *versus* clustered QDs), for example Crooker *et al.*^42^ found a red-shift of 8 nm (35 meV) for CdSe nanoclusters, which is smaller than the red-shift of 45 nm (122 meV) observed for our CuInS_2_/ZnS QD nanoclusters. The significantly larger red-shift observed in the current study is likely to arise from either the different structure of the assembly (different packing density of QDs), or from the increased size dispersion of individual QDs (18% in the current study *versus* 7% dispersion for Crooker *et al.*) which would lead to a significantly larger energy difference between the smallest and largest QDs within each structure.

### 3.6 Future outlook for lipid-stabilized QD clusters

In the current work, our assembly procedure was demonstrated to successfully stabilize tetrahedral CuInS_2_/ZnS QDs with lipids, but there are still other structural aspects to explore. For example, the impact of QD shape on their encapsulation by lipids has not yet been explored. Spherical QDs, in contrast to tetrahedral ones, may exhibit different lipid interactions due to fewer contact points between lipid tails and QD capping ligands. This could affect QD structure and aggregation when combined with lipids. For some applications it might be preferable to generate individual hydrophobic QDs incorporated within a lipid bilayer as opposed to clustered QDs surrounded by a lipid monolayer. While achieving both efficient FRET and insertion of QDs within the centre of a lipid bilayer at high concentrations may be challenging,^64^ using spherical QDs may reduce the size of QD clusters, if this is desired. Additionally, switching to different types of QDs will alter their optical properties. CuInS_2_/ZnS QDs typically have broad emission bands which represent a population of QDs of different sizes, leading to the large red-shift observed due to QD-to-QD FRET.^44,45^ If a different sample of QDs that had a narrower population distribution of sizes was used then this would result in less of a red-shift. We could also consider QD optical properties at the single-particle level (not measured in the current study).^76,79^ If individual QDs have a narrower emission spectrum and with the knowledge that different chemistries of QDs are known to have different peak widths then this will change the light harvesting properties of nanocluster assembled from alternative QD types.^42^ QDs having very narrow emission bands on the single-particle level can be expected to have a lower efficiency of QD-to-QD energy transfer due to a lower probability of energetic overlap to the next-nearest QD particle in a cluster. Further investigations, including TEM imaging correlated to time-resolved fluorescence measurements of single particles, could quantify how QD size influences the structure and optical properties of self-assembled QD nanoclusters in even greater detail. Understanding this relationship might enable precise control over the energy transfer pathways and photoluminescence of future nanoclusters of QDs. Improved control of how to interface QDs with biomolecules will support the next generation of biosensors^80–85^ and designing “bio-hybrid” and “bio-inspired” materials with specific physical properties,^86,87^ such as coupling QDs to proteins from biological photosynthesis^88–92^ or interfacing QDs with living microbial cells.^93–96^

## 4 Conclusions

In summary, we have demonstrated a new procedure that utilizes the self-assembly of lipids to stabilize small clusters of hydrophobic QDs in aqueous solution. This procedure is suitable for low-toxicity CuInS_2_/ZnS QDs. Our comprehensive analysis of the structural and optical properties of these QD–lipid nanoclusters contributes to our fundamental knowledge of how QD–QD and QD–molecule physical associations can change their photophysical interactions. Electron microscopy revealed that these assemblies had a core of clustered QDs with diameters of approximately 40 nm. Particle sizing measurements and fluorescence microscopy showed that there were larger lipid-only assemblies or, potentially, higher-order lipid/QD assemblies of 100–200 nm in hydrodynamic size. Perhaps, any unwanted lipid-only assemblies could be removed by protocol optimization or further purification steps in future work. Fluorescence microscopy validated the co-localization of lipids and QDs, confirming their association and the role of lipids in stabilizing hydrophobic QDs in aqueous environments. The threshold quantity of lipid required during the assembly procedure to stabilize these clusters of QDs was determined as a ratio of 1250 lipids per QD, likely representing the saturation of available QDs in the presence of excess lipid. Spectroscopic analysis revealed the photophysical properties of the QD nanoclusters, including a pronounced red-shift in fluorescence emission of nanoclusters compared to individual QDs that was attributed to Förster Resonance Energy Transfer from higher-energy (smaller) QDs to lower-energy (larger) QDs, consistent with previous studies on QD aggregates. These findings show that the assembly of QD nanoclusters can be controlled by lipids and this hybrid system can act as a platform for controlled energy transfers, with the potential for tunability based on QD size, shape, and composition. Future studies exploring different QD chemistries, narrower size distributions, and single-particle fluorescence measurements may further optimize these systems for potential applications in biosensors or other biohybrid systems.

## Author contributions

Joel Whipp: investigation (QD synthesis, QD–lipid assembly, all spectroscopy, epifluorescence microscopy), formal analysis, methodology, validation, data curation, visualization, writing – original draft, writing – review & editing; Ashley Hancock: investigation (fluorescence lifetime imaging microscopy), formal analysis, validation, visualization, writing – review & editing; Zabeada Aslam: investigation (electron microscopy), formal analysis, writing – review & editing; Kevin Critchley: conceptualization, supervision, funding acquisition, writing – review & editing; Peter Adams: visualization, conceptualization, supervision, funding acquisition, writing – original draft, writing – review & editing.

## Conflicts of interest

There are no conflicts of interest to declare.

## Supplementary Material

NR-018-D6NR00297H-s001

## Data Availability

All relevant raw and analyzed data associated with this paper are openly available under a CC-BY license in the Research Data Leeds repository^97^ at https://doi.org/10.5518/1725. Supplementary information (SI) is available. The SI document contains additional experimental data and associated explanations: (i) control samples showing the importance of lipid and detergent in the procedure to form QD nanoclusters, (ii) particle sizing of QD-lipid assemblies (NTA and zeta potential measurements), (iii) fluorescence excitation spectra of QD-lipid assemblies, (iv) further electron microscopy images and analyses of colloidal QDs and QD-lipid nanoclusters (TEM and STEM), (v) further information and images related to FLIM data on QD-lipid assemblies, (vi) fitting of fluorescence decay curves, (vii) further data on the photophysics of QD-lipid assemblies (fluorescence excitation versus emission plots). See DOI: https://doi.org/10.1039/d6nr00297h.
