# Supplementary material for: Analysis of lipid-assisted self-assembly of hydrophobic CuInS2/ZnS quantum dots into water-stable nanoclusters that perform intra-cluster energy transfer
Source: Nanoscale. 2026 May 20;18(24):12942–57. doi: 10.1039/d6nr00297h (PMC13189225; doi:10.1039/d6nr00297h)
Supplement: NR-018-D6NR00297H-s001 [file NR-018-D6NR00297H-s001.pdf]

Supplementary Information (SI) for Nanoscale.
This journal is © The Royal Society of Chemistry 2026

Supplementary Information for:

Analysis of lipid-assisted self-assembly of hydrophobic $CuInS_2/ZnS$ quantum dots into water-stable nanoclusters that perform intra-cluster energy transfer

Joel T. Whipp,[a,b] Ashley M. Hancock,[a,b‡] Zabeada Aslam,[c] Kevin Critchley*[a] and Peter G. Adams*[a,b]

[a] School of Physics and Astronomy, University of Leeds, Leeds LS2 9JT, UK; E-mail: k.critchley@leeds.ac.uk and p.g.adams@leeds.ac.uk

[b] Astbury Centre for Structural Molecular Biology, University of Leeds, Leeds LS2 9JT, UK

[c] Leeds Electron Microscopy and Spectroscopy Centre, School of Chemical and Process Engineering, University of Leeds, Leeds LS2 9JT, UK

‡ Current address: ICFO-Institut de Ciencies Fotoniques, The Barcelona Institute of Science and Technology, Castelldefels, Barcelona 08860, Spain

Control samples showing the importance of lipid and detergent in the procedure to form QD nanoclusters

To assess the significance of lipids and detergents in the nanocluster self-assembly process, control experiments were conducted with individual components omitted. Our understanding of the procedure is outlined below, with reference to the schematic from the main text and spectra reported below:

(i) In the first stage of the procedure, QDs and lipids (where included) are mixed in chloroform, in the desired ratios (main text **Figure 1**, *panel 1*). Absorbance spectra of $CuInS_2$/ZnS QDs in chloroform were identical both with and without lipids included, indicating that the nanoparticles were undisturbed by the presence of lipids when the solvent was chloroform (**Figure S1A**, *black* vs. *red* line). The fluorescence emission spectra and fluorescence decay curves are also very similar (**Figure S1B** and **C**, *black* vs. *red* lines) with just a 13% decrease in intensity and a very slightly faster decay attributed to the presence of lipids. This all suggests that the lipids have no significant effect on the optical properties of the QDs when in chloroform (i.e., the optical changes observed at later stages of the protocol related to QD/lipid self-assembly require an aqueous environment).

(ii) In the second stage, the chloroform is removed by evaporation to dry the QDs (and lipids) into a film on the inside of a glass vial (main text **Figure 1**, *panel 2*). It was not possible to acquire spectra from this stage of the process.

(iii) In the third stage, the QD/lipid film is rehydrated by an aqueous buffer solution (main text **Figure 1**, *panel 3*). The optimal procedure included detergent (4% sodium cholate) in this buffer and this resulted in an absorbance spectrum with a relatively similar shape to QDs in chloroform (**Figure S1A**, *lilac* vs. *red* line). This suggested that the QDs were nicely resuspended. However, the fact that the absorbance curve appeared to be translated vertically to higher values than the curve in chloroform suggested scattering was occurring which could relate to the formation of larger particles which could include QD clusters (main text **Figure 1**, *panel 4*). This idea is supported by a quenching (by about 40%) of the fluorescence intensity observed in emission spectra (**Figure S1B**, *lilac* vs. *red* line) and the significantly faster fluorescence decay (**Figure S1C**, *lilac* vs. *red* line). A 45-nm red-shift of the peak wavelength was also evident in the emission spectrum. This all suggests that there could be energy transfer between QDs, as discussed later in the main text.

(iv) If the QD/lipid film was rehydrated with an aqueous buffer solution that did *not* include any detergent then the absorbance spectrum showed a much higher level of scattering, appearing at even higher "apparent absorbance" values (**Figure S1A**, *blue* vs. *lilac* line). The features of the absorbance spectrum related to QDs such as the first excitation transition were poorly defined. Interestingly, the fluorescence decay curve was very similar with and without detergent (**Figure S1C**, *blue* vs. *lilac* line) but the fluorescence intensity was much lower (**Figure S1B**, *blue* vs. *lilac* line) which could suggest that there was a similar sort of energy transfer process was occurring but that only half as many quantum dots remained. It seemed likely that uncontrolled agglomeration of the nanoparticles had occurred.

(v) In the final stage of the procedure, Biobeads are used to remove the detergent from the QD/lipid/detergent mixture (main text **Figure 1**, *panel 5*). The presence of lipids at this stage was crucial to maintain the solubility of the QDs. Without lipids there was minimal absorbance or fluorescence remaining after detergent removal (**Figure S1A-B**, *green* line). This suggested that QDs had precipitated out of solution and been lost. With lipids, the absorbance features were mostly maintained including the shape of the curve with a vertical shift downwards, as compared to before detergent removal (**Figure S1A**, *yellow* vs. *lilac* line). The fluorescence decay curve was even steeper (**Figure S1C**, *yellow* vs. *lilac* line) and the fluorescence intensity was 50% lower (**Figure S1B**, *yellow* vs. *lilac* line). Altogether, this data suggested that there was even more energy transfer occurring and that the majority of quantum dots remained, due to the stabilizing effect of the lipids.

(vi) The possible structures that may be generated at the end of the process are suggested in main text **Figure 1**, *panels 6A-6D*, however, they cannot be distinguished using spectroscopy alone. Further structural and optical characterization of these end-point samples is a major subject of the main text.

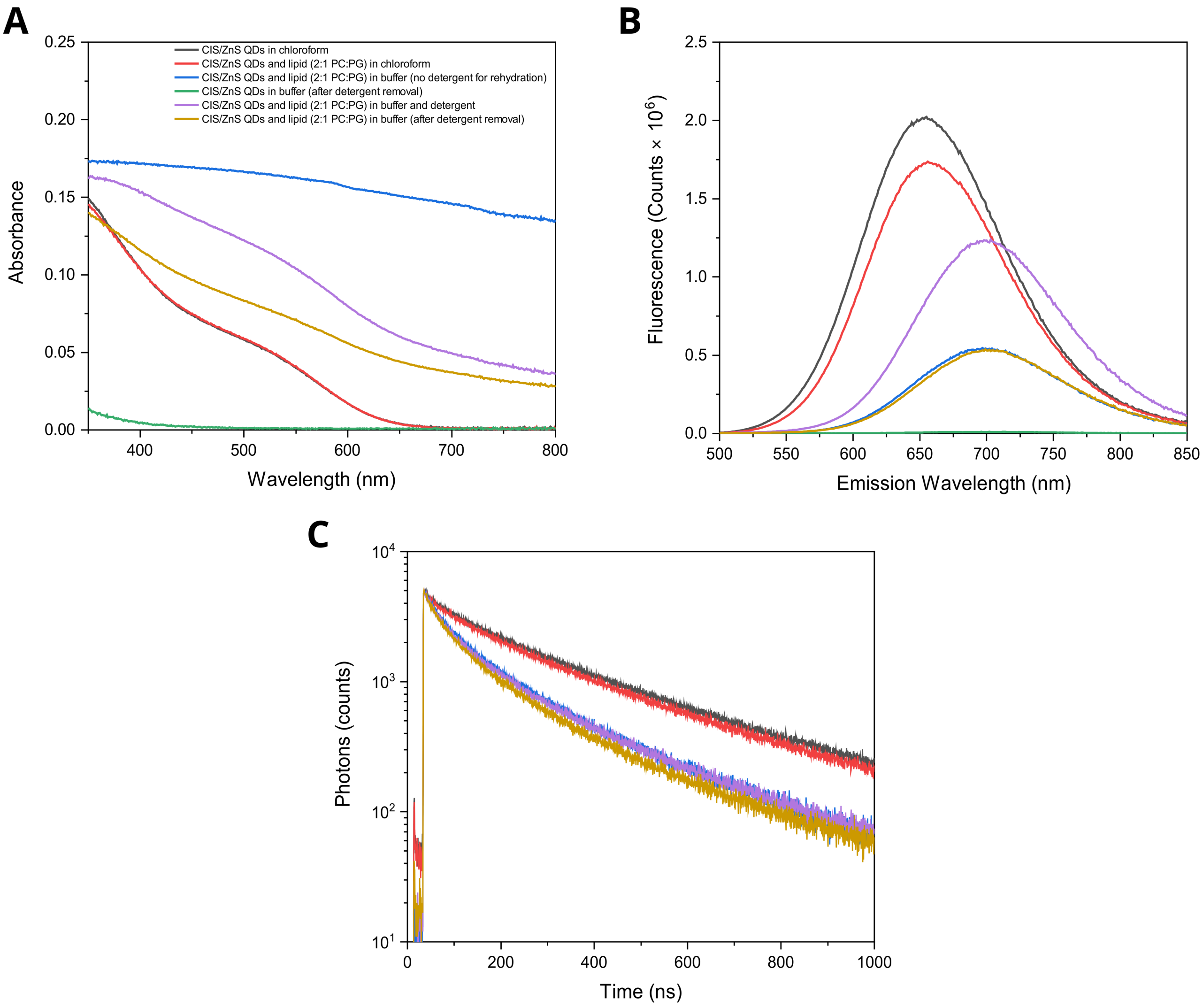

Figure S1: Spectra of $CuInS_2$ QDs at each stage of the nanocluster self-assembly procedure. (A) Absorbance spectra of the sample series of QDs and the associated lipids (if included) at each stage of the procedure, with negative control measurements included. (B) Steady-state fluorescence of the same samples as in (A). (C) Fluorescence decay curves, of the same samples as in (A), acquired by time-correlated single-photon counting measurements.

Initial particle sizing of QD-lipid assemblies

Nanoparticle Tracking Analysis (**Figure S2**) and zeta potential measurements (**Figure S3**) to provide an initial assessment of the particle size.

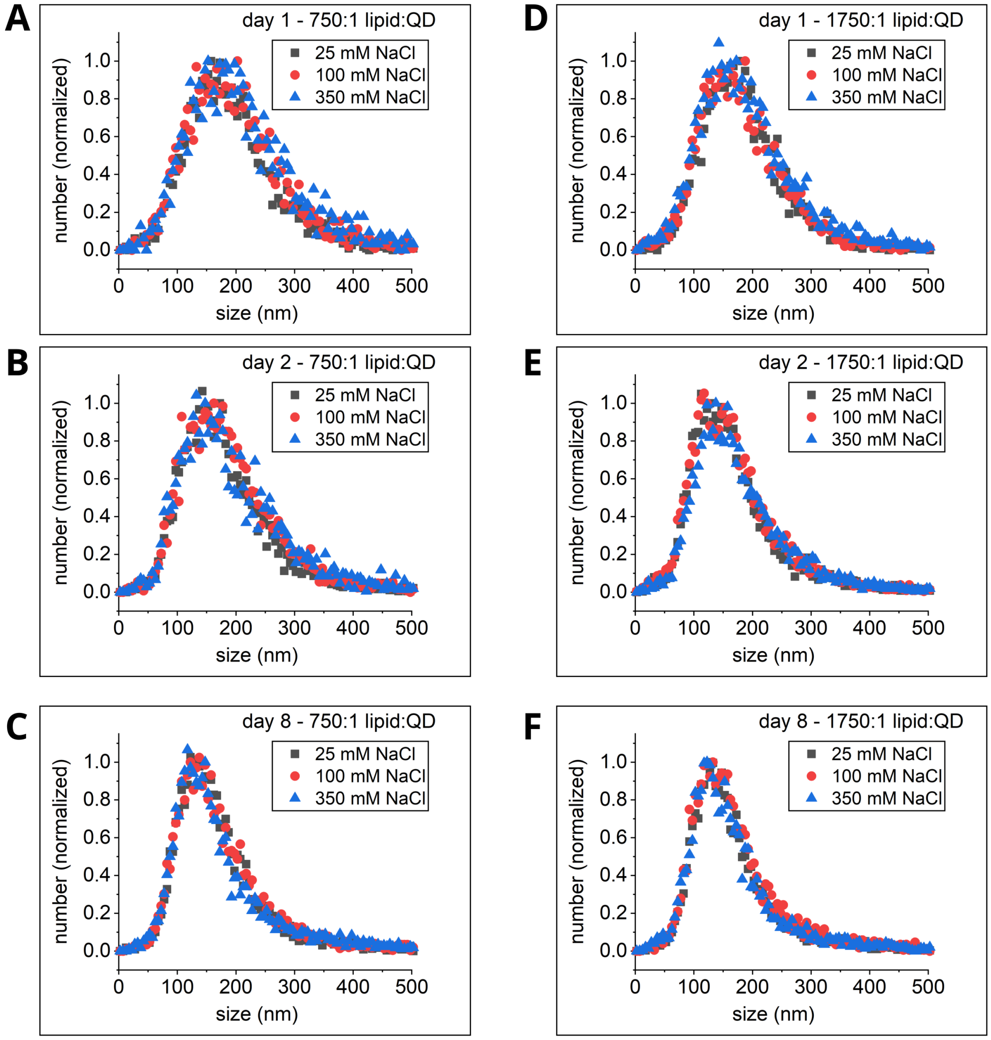

Figure S2: Nanoparticle Tracking Analysis (NTA) comparing QD-lipid assemblies at various timepoints and salt concentrations. NTA data for clusters assembled with 750:1 lipid:QD measured on (A) day 1, (B) day 2 and (C) day 8. NTA data for clusters assembled with 1750:1 lipid:QD measured on (D) day 1, (E) day 2 and (F) day 8. A single sample was prepared for each lipid:QD ratio using an aqueous buffer of 50 mM HEPES, 100 mM NaCl, pH 7.5 and then split into three different buffers for measurements and storage, either: (i) 12.5 mM HEPES, 25 mM NaCl, pH 7.5, (ii) 50 mM HEPES, 100 mM NaCl, pH 7.5, (iii) 100 mM HEPES, 350 mM NaCl, pH 7.5. Samples were stored at 4 degrees in the dark in between measurements.

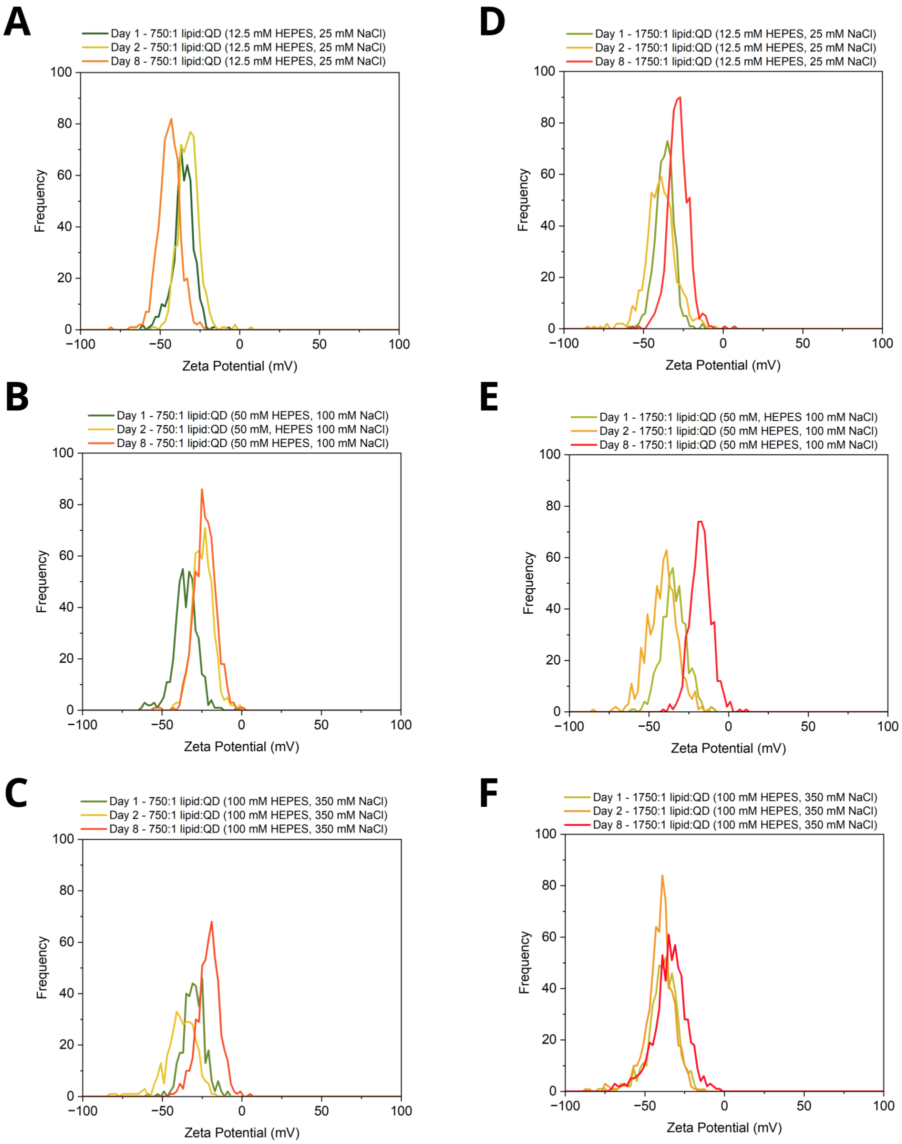

Figure S3: Zeta Potential measurements comparing QD-lipid assemblies at various timepoints and salt concentrations. The same samples as in the NTA analysis were measured, in the aqueous buffers noted. All samples have a similar zeta potential of approx. -40 mV on day 1. For some samples, the zeta-potential is reduced by day 8, but this is not consistent.

Fluorescence excitation spectra of QD-lipid assemblies

Absorbance spectra (main text **Figure 2A**) can have artifacts due to light scattering. Therefore, fluorescence excitation spectra were also acquired which do not typically have scattering effects. These spectra showed that the shape of the curves and the position of the first excitation peak (500-600 nm) was similar whether the QDs were isolated or clustered (**Figure S4A**). There were subtle changes in spectral shape when the monitoring wavelength was changed but the agreement between 650-700 nm was excellent (**Figure S44B-D**). The greater intensity obser ved for the clustered QDs at the higher wavelength range (350-550 nm) may relate to the effect of energy transfer between QDs that occurs in the clustered system.

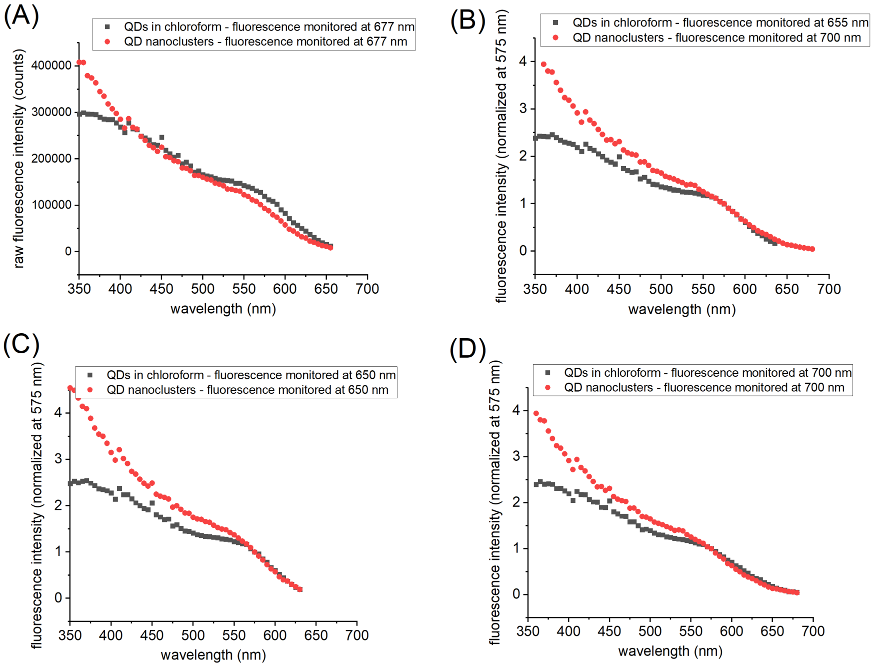

Figure S4: Fluorescence excitation spectra comparing isolated QDs with clustered QDs. A series of graphs comparing the spectra but with monitoring of the fluorescence signal at different wavelengths. (A) Spectra where fluorescence was monitored at the same wavelength, 677 nm, intermediate between the emission maxima. The raw counts are shown on this graph. (B) Spectra where fluorescence was monitored at different wavelengths: the peak for each sample (655 nm emission peak for QDs in chloroform, 700 nm emission peak for QDs). The spectra were normalized to 1.0 at 575 nm to allow comparison of graph shapes. (C) Spectra where fluorescence was monitored at the same wavelength, 650 nm, which is preferential for the emission of QDs isolated in chloroform. Spectra were normalized to 1.0 at 575 nm, as in panel B. (D) Spectra where fluorescence was monitored at the same wavelength, 700 nm, which is preferential for the emission of clustered QDs. Spectra were normalized to 1.0 at 575 nm, as in panel B.

Electron microscopy of colloidal QD

The resulting QD-lipid structures underwent transmission electron microscopy (TEM) and scanning-transmission electron microscopy (STEM) measurements to determine the size of the aggregates. Prior to TEM analysis, the size of an individual QD was estimated based on previous studies correlating size with absorption properties. Both colloidal $CuInS_2$/ZnS QDs and lipid-stabilized QD nanoclusters were prepared at a concentration of 6.81 µM, calculated using the Beer-Lambert law ($A = \varepsilon Cl$) with a molar absorption coefficient derived from a published empirical equation [1]. A fluorescence emission peak at 655 nm (see main text **Figure 2**) yields a theoretical average QD size of 2.71 nm for the ensemble, with a molar absorption coefficient of 93,490 M^{-1} cm^{-1}. This theoretical QD size serves as a reference point for comparison with the average size determined through TEM analysis.

To ascertain the average size of $CuInS_2$/ZnS QDs, both semi-automated and manual analysis of STEM and TEM images was conducted using ImageJ. First, the size of individual (colloidal) QDs was assessed on samples of $CuInS_2$/ZnS QDs that were deposited (drop cast) onto graphene/carbon-coated Cu grids by TEM imaging (main text **Figure 2D-E** and **Figure S5**). Manual size analysis posed challenges due to the low contrast of the TEM, making it difficult to accurately discern the thin QD boundaries from background noise, as shown in the example image in **Figure S6A**. Consequently, this might lead to particle size measurements smaller than those obtained from semi-automated analysis using high-contrast STEM, although it should provide a reliable estimate of the size of the electron-dense core of QDs. The size was defined as the longest corner-to-side distance of the triangular projection, and examples of the lengths drawn manually in ImageJ are shown in **Figure S6B**). The distribution resulting from manual size analysis is depicted in **Figure S6C**. The average core size of the $CuInS_2$/ZnS QDs was determined to be 2.9 ± 0.4 nm, slightly surpassing the theoretical estimations for the ensemble average QD core size of 2.71 nm calculated using the molar absorption coefficient [1]. This is reasonable because the theoretical (2.71 nm) size does not include the ZnS shell structure which will increase the size observed by TEM. Additionally, a size dispersion, expressed as the standard deviation as a percentage of the mean, was found to be 13%.

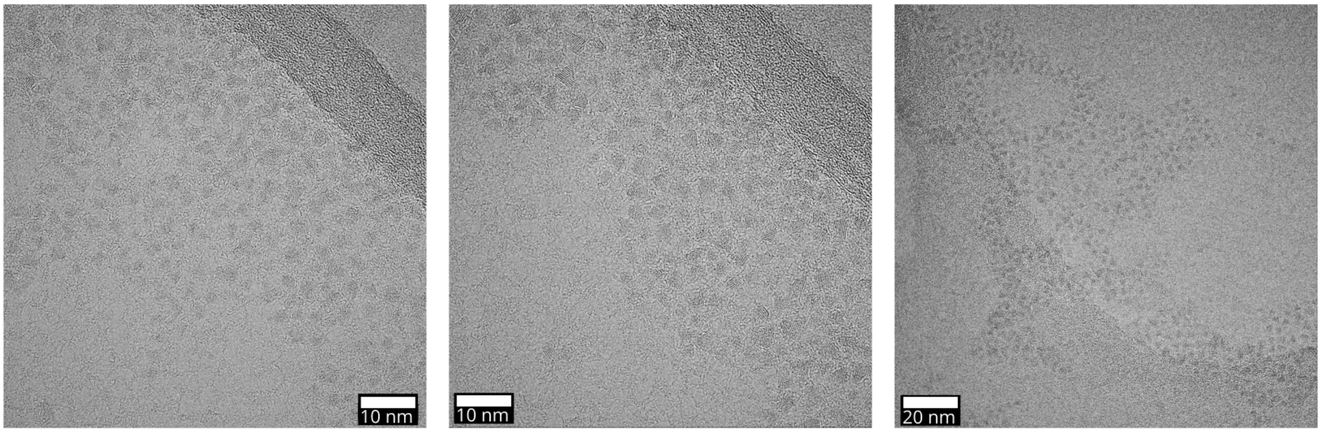

Figure S5: Gallery of further TEM images of colloidal $CuInS_2$/ZnS QDs. Scale bars are shown.

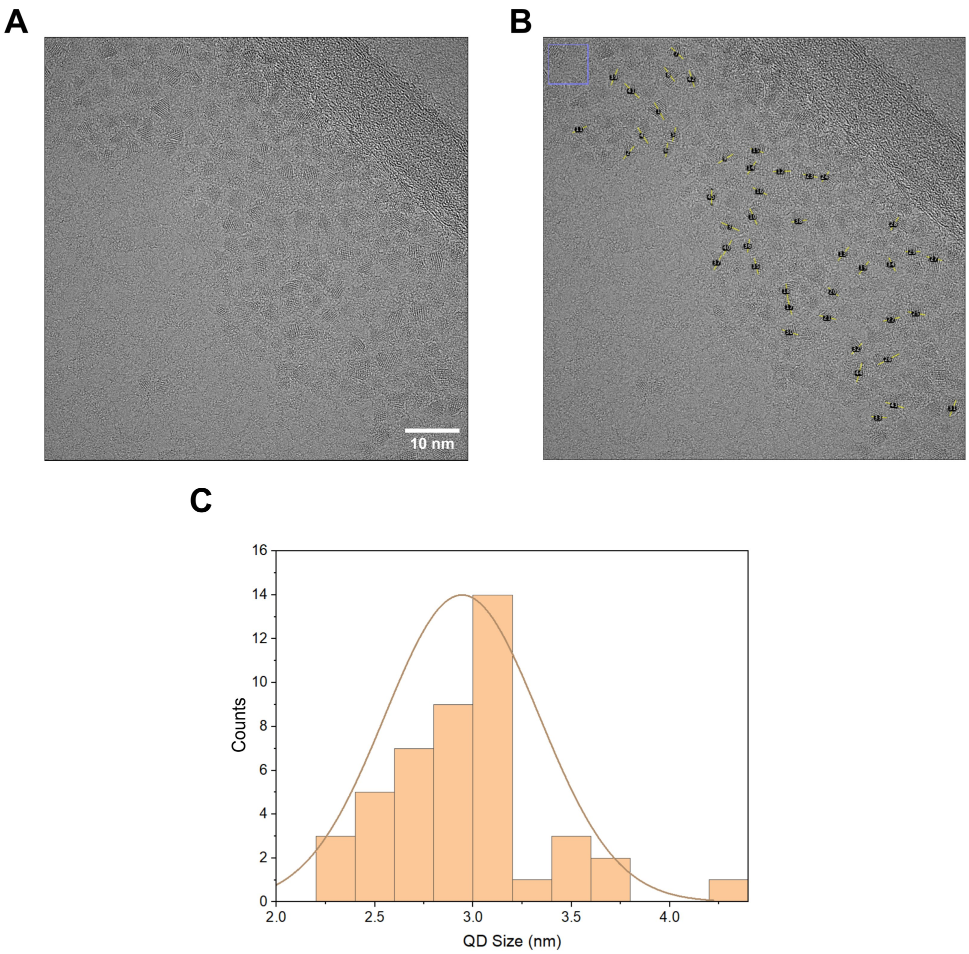

Figure S6: Size distribution of colloidal $CuInS_2$/ZnS QDs following manual analysis. (A) Example of original TEM image used for manual size analysis of QDs that had been dried onto a Cu grid coated with a carbon film and graphene oxide. The scale bar is 10 nm. (B) Example of manual size analysis on individual QDs, using the TEM image from (A) and showing annotations where the length was measured. (C) Histogram showing the size distribution of QDs from the manual analysis. Bin widths are 0.2 nm, mean size = 2.9 ± 0.4 nm.

To validate the representativeness of the determined size, a comparison was made between the analysis of TEM data conducted on a smaller particle subset and the semi-automated size analysis of STEM data. While STEM offers enhanced contrast compared to TEM, it suffers from lower spatial resolution so it was useful to compare the findings from the two different techniques. **Figure S7** presents a series of STEM images featuring colloidal tetrahedral $CuInS_2$/ZnS QDs deposited on graphene-coated grids at various magnifications. The tetrahedral morphology and size distribution are more distinctly observed in these STEM images compared to TEM, owing to the high contrast between the atomically-dense QDs and the background. Notably, visible groupings of QDs are discernible, suggesting a close-packed arrangement on the surface (**Figure S7A-B**). Furthermore, QDs exhibit association at grid boundaries (**Figure S7C-D**), consistent with the observations made from TEM analysis, as previously noted.

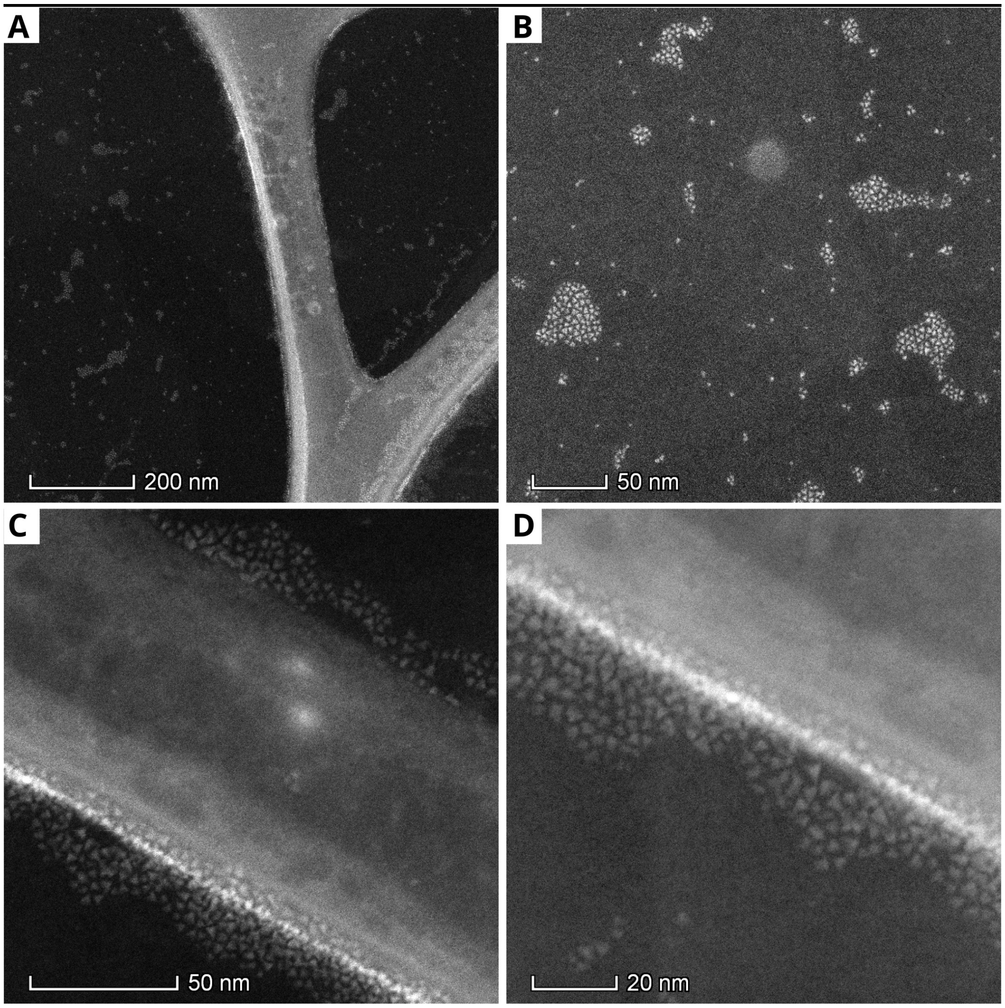

Figure S7: Example STEM images of colloidal $CuInS_2/ZnS$ QDs that were used for semi-automated size analysis. Scale bars are (A) 200 nm, (B) 50 nm, (C) 50 nm, and (D) 20 nm.

Owing to the pronounced contrast between the QDs and the grid, semi-automated analysis could be conducted on the STEM images, enabling comparison with manual size analysis performed on the TEM images. The determination of $CuInS_2/ZnS$ QD "size," referring again to the longest corner-to-side distance of the triangular projection in TEM/STEM, commenced with initial calibration of the scale using the ImageJ scale bar (**Figure S8A-B**). Subsequently, the threshold for size analysis was established (**Figure S8C**), facilitating identification of individual particles for automated size analysis due to their high contrast with the background (inverted images shown in **Figure S8D**). As depicted in **Figure S8E**, the applied threshold failed to segregate the colloidal QDs that had dried adjacently, necessitating the application of a "watershed function" to delineate the boundary around each individual particle. The resulting particle selection is shown in **Figure S8 F**, consistently applied across all captured STEM images containing colloidal $CuInS_2/ZnS$ QDs. A data selection parameter was implemented to exclude artifacts based on area, followed by manual removal of "extreme particles" that did not represent QDs but were not omitted by the software. The resulting size distribution, displayed in **Figure S8G**, conforms well to a Gaussian normal function. The average size was computed to be 3.3 ± 0.6 nm, exceeding the size obtained from TEM images (2.9 ± 0.4 nm), indicating a size dispersion of 18%. This dispersion is slightly larger than that calculated from manual analysis of TEM images (13%). The significant dispersion may indicate some variability during shell growth or core concentration or there may be variability resulting from cation exchange during shell formation.

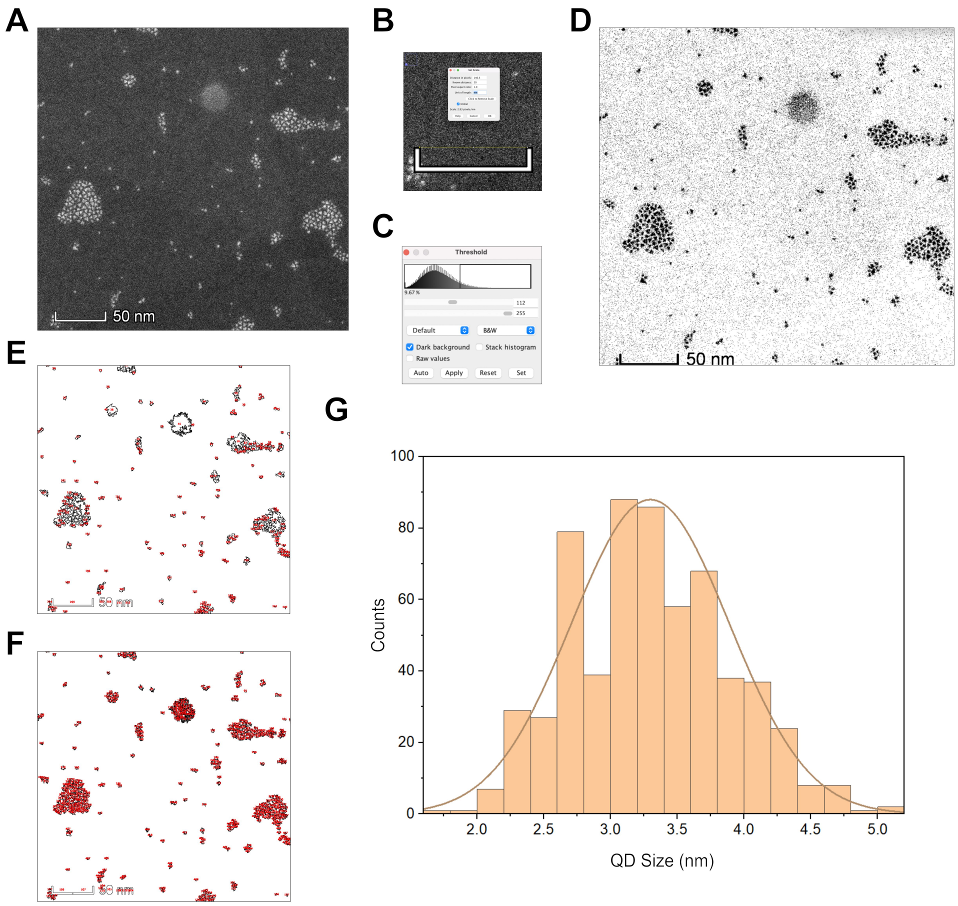

Figure S8: Automated analysis of colloidal $CuInS_2/ZnS$ QD size. (A) Selected STEM image used for automated nanoparticle size analysis in ImageJ. Scale bar 50 nm. (B) Calibration of the scale for nanoparticle size analysis. (C) Threshold designation for nanoparticle selection. (D) The resulting contrast-enhanced image that allows nanoparticle selection after applying a threshold. (E) Initial particle selection from original threshold parameters. (F) Final particle selection after applying a watershed function to separate boundaries of large clusters. (G) Histogram showing the size distribution of QDs from the semi-automated analysis. Bin widths are 0.2 nm, mean size = 3.3 ± 0.6 nm.

The smaller distribution obtained from manual analysis may also be attributed to a smaller sample size, with 47 particles analyzed manually compared to hundreds counted during semi-automated analysis. The obtained size and relatively wide dispersion of the QDs align with expectations for tetrahedral $CuInS_2/ZnS$ QDs synthesized using identical or similar techniques [2–4].

Electron microscopy of QD-lipid nanoclusters

TEM images of QD-lipid nanoclusters are described in the main text, with a further gallery of images shown in Figure S9. Images of QD-lipid nanoclusters acquired with STEM, a technique which provides higher contrast, are shown below in **Figure S10**. Due to the overalpping QD particles the intensity at the centre of the cluster of QDs is higher than at the periphery of the clusters. There is no spacing or hexagonal packing of QDs, as was observed in the STEM images of colloidal QDs (**Figure S7**), due to the 3-D nature of the clustered QDs.

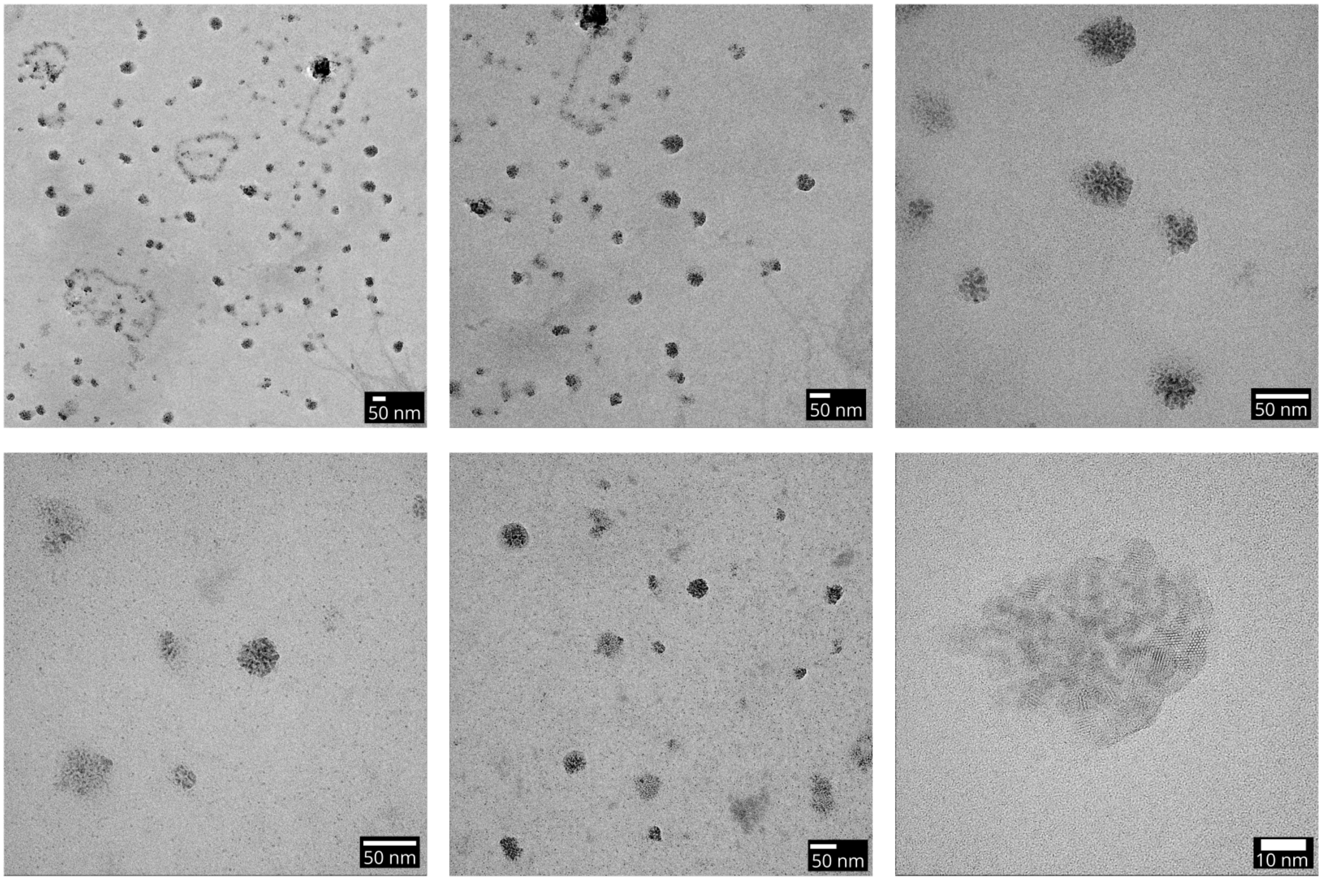

Figure S9: Gallery of further TEM images of QD-lipid nanoclusters. Scale bars are shown.

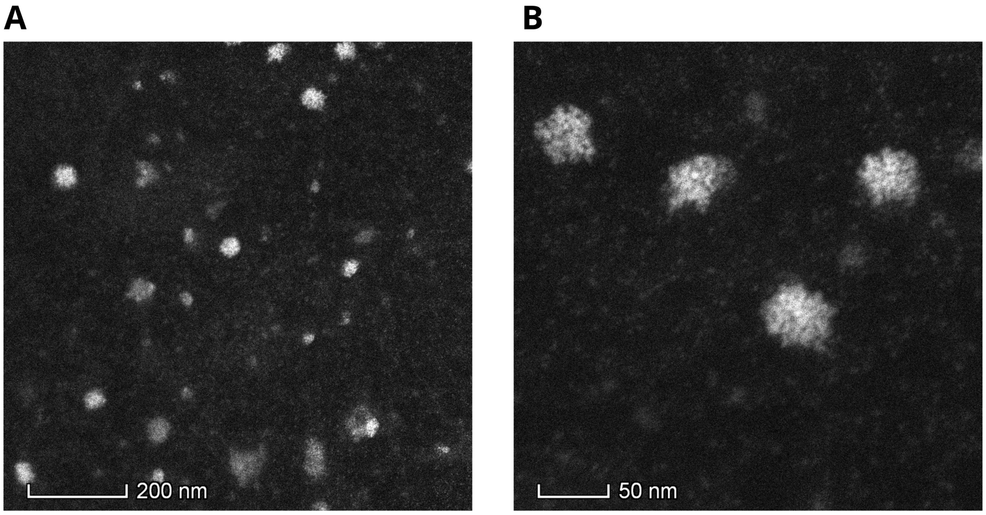

Figure S10: STEM images of nanoclusters of $CuInS_2$/ZnS QD and lipids prepared in aqueous buffer and then deposited onto grids. Scale bars are (A) 200 nm, (B) 50 nm.

Fluorescence Lifetime Imaging Microscopy

Amplitude-weighted mean lifetime calculations were conducted using SymPhoTime software (PicoQuant), involving the generation of fluorescence decay curves from the accumulated photons within a designated region-of-interest (ROI) within an image, followed by fitting the curve to a multi-exponential decay function. To illustrate this process, an example is presented in **Figure S11** . **Figure S11A** exhibits a composite of both the NBD and QD fluorescence, in an image measuring 5 × 5 µm, showing a QD nanocluster. In **Figure S11B**, the QD channel, manually selected from the composite image, is showcased to pinpoint the QD signal spatially coinciding with the signal acquired from the QDs. **Figure S11C** displays the lifetime histogram derived from the pixels within the ROI drawn around the QD signal calculated utilizing the SymPhoTime software.

To quantify the most representative value for the lifetime of QD-lipid nanoclusters, images were analyzed where there was a field containing several individual clusters that could be identified. Amplitude-weighted lifetimes were computed from this sub-population of nanoclusters through a series of steps. Initially, thresholding was applied to images to eliminate particles with insufficient fluorescence for further analysis, leaving behind particles suitable for individual analysis. The threshold, set to exclude pixels registering below 10 counts, ensured robust selection of particles. Subsequently, single particles were meticulously chosen, with careful attention to avoid overlapping nanoclusters. Regions-of-interest (ROIs) were manually delineated around these individual particles using the "paint ROI" function within the SymPhoTime software. Following ROI selection, time-correlated single-photon counting (TCSPC) data from each ROI were subjected to a bi-exponential re-convolution fit employing a measured instrument response function (IRF). The IRF was acquired from a glass slide under identical excitation laser and objective lens conditions. Fits with a χ^2 value exceeding 1.5 were excluded from statistical analysis to ensure data quality. The process for determining amplitude-weighted lifetimes for NBD-DHPE samples mirrored that of the QD nanoclusters, albeit without the need for initial thresholding due to the inherent fluorescence characteristics of the samples (higher signal).

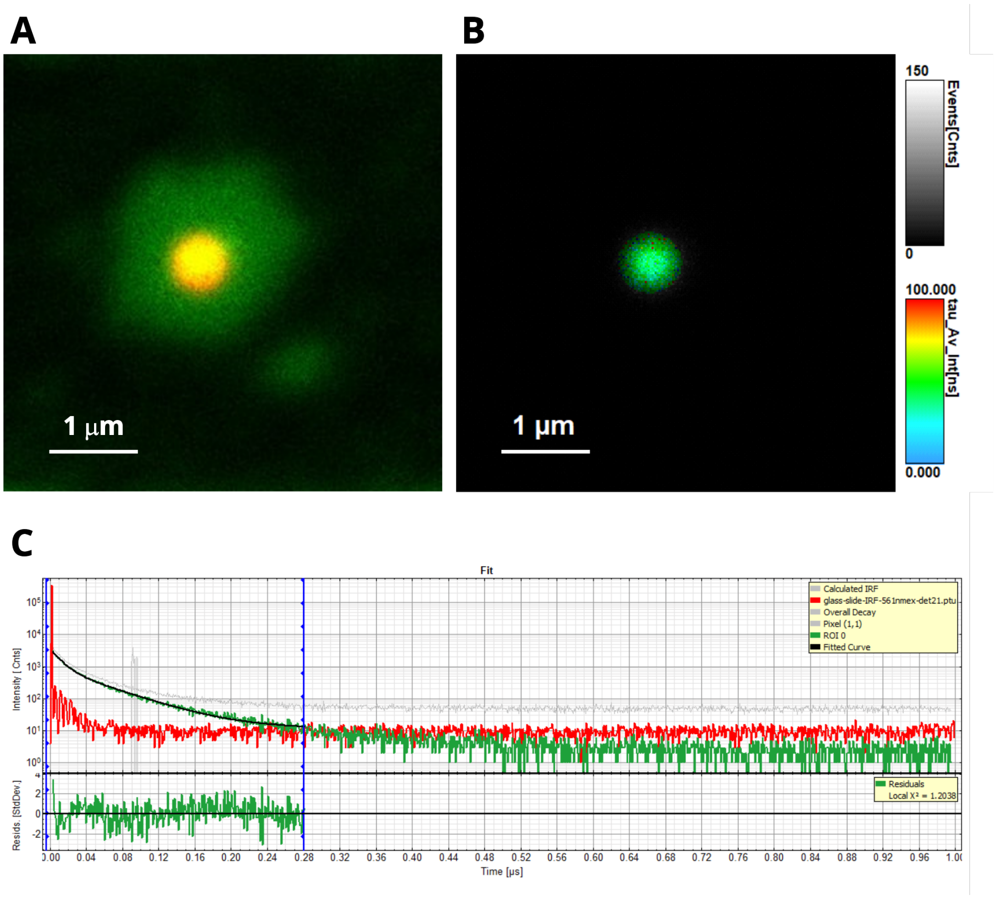

Figure S11: Example of lifetime determination from captured FLIM images. (A) Original composite image of a 33:66:1 DOPG:DOPC:NBD-DHPE-coated QD nanocluster with the QD fluorescence shown in *red*, the NBD-DHPE fluorescence shown in *green*, and spatial overlap between the NBD-DHPE and QDs shown in *yellow*. (B) FLIM image showing the intensity and lifetime in a selected region-of-interest from the QD channel from (A). (C) The resulting lifetime trace and fit taken within SymPhoTime software.

Figure S12 presents illustrative images showcasing the particle selection process. Each particle's color corresponds to its amplitude-weighted lifetime relative to the upper limit of the FLIM false-color scale (10 ns for **B** and **D**), while brightness reflects fluorescence intensity. Three distinct samples were prepared: (i) a QD-lipid nanocluster sample devoid of the fluorescent lipid NBD-DHPE, (ii) an lipid vesicle sample that contained NBD-DHPE probes, devoid of any QDs, and (iii) a mixed QD-lipid-NBD nanocluster sample. Fluorescence from QDs and NBD was collected in separate channels. In **Figure S12A**, ROI selection for the QD-only sample is depicted, with five larger clusters chosen, each yielding a sufficient number of counts to extract the lifetime of QDs. **Figure S12B** showcases ROI selection for the liposome/NBD-DHPE sample, utilized for control measurements to determine the usual lifetime of NBD. **Figures S12C** and **D** display ROI selection for the QD channel and NBD channel, respectively, in the QD/NBD-lipid mixed sample. For this mixed sample, ROIs were initially chosen for in the QD channel, with identical ROIs employed to calculate the lifetime of co-localized NBD in corresponding areas. This facilitated the observation of any potential reduction in NBD lifetime that could be attributable to energy transfer from NBD to QDs.

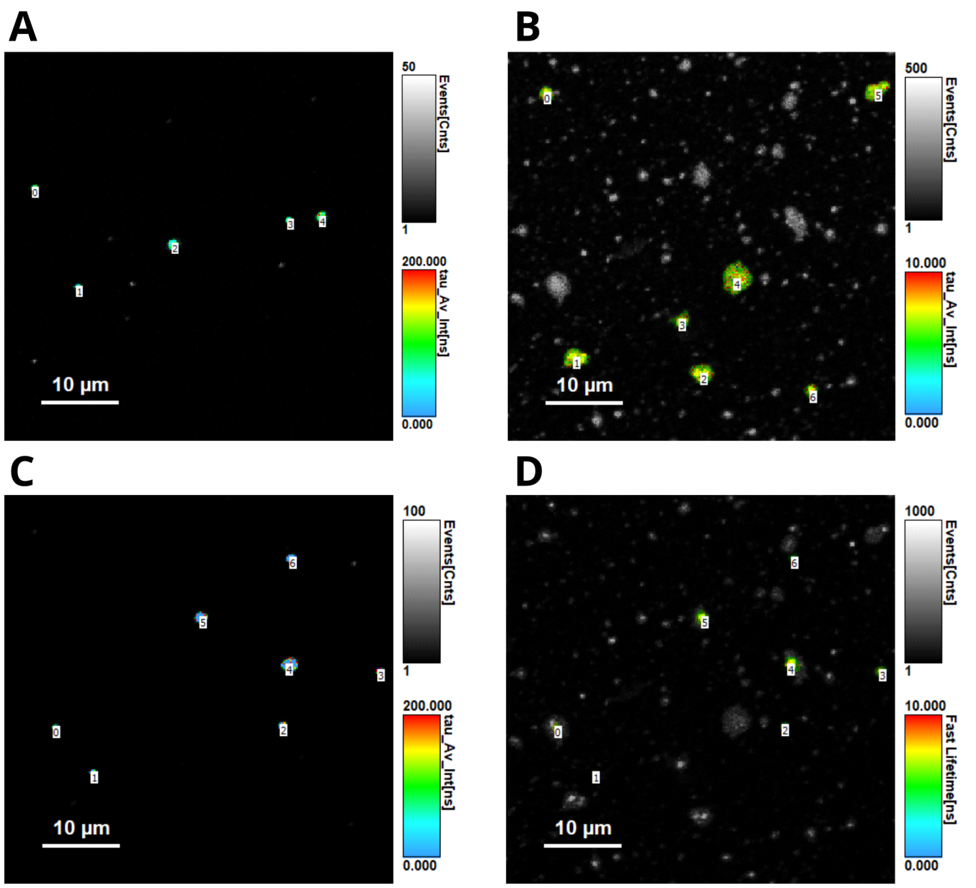

Figure S12: FLIM images showing both fluorescence intensity and lifetime data of selected regions of interest. (A) ROI selection to determine the lifetime of QD-lipid nanoclusters (no NBD lipids) in the QD channel. These QD-lipid nanoclusters were prepared using a lipid mixture of 1:2 DOPG:DOPC and lipid:QD molar ratio of 1500:1. (B) ROI selection to determine the lifetime of NBD-DHPE within lipid vesicles (no QDs) in the NBD channel. The lipid ratio in this mixture was 33:66:1 DOPG:DOPC:NBD-DHPE. (C) This panel displays the QD channel with the ROI selection used to determine the lifetime of QDs within the QD-lipid nanoclusters, in the presence of the NBD-DHPE lipid. These QD-lipid nanoclusters were prepared using a lipid mixture of 33:66:1 DOPG:DOPC:NBD-DHPE and lipid:QD molar ratio of 1500:1. (D) This panel displays the NBD channel for the same ROI as panel (C). All samples were deposited on a glass substrate and imaged under the standard aqueous buffer solution.

The amplitude-weighted average lifetimes derived from the ROIs were aggregated into a histogram and a scatter plot, as depicted in **Figure S13**. The frequency distribution of NBD-DHPE lifetimes, in **Figure S13A**, reveals a noteworthy quenching of NBD fluorescence induced by the QDs. Specifically, the mean NBD fluorescence lifetime diminishes from 3.40 ± 0.45 ns (in the absence of QDs) to 2.58 ± 1.09 ns upon encapsulation of QD nanoclusters. This reduction in NBD fluorescence lifetime provides good evidence of Förster Resonance Energy Transfer (FRET) from NBD to QDs. Such a phenomenon underscores the proximity of a significant amount of lipids to QDs, necessary for FRET to manifest. The Efficiency of Energy Transfer (ETE) can be estimated from the amplitude-weighted lifetime of NBD using the following equation:

$$ETE = 1 - \frac{\tau_{DA}}{\tau_{f,D}} \tag{1}$$

where τ_{DA} and $F_{f,D}$ are the mean fluorescence lifetimes of NBD in the presence or absence of the QDs, respectively.

An energy transfer efficiency (ETE) of 24.1 ± 10.7% was determined for NBD-to-QD FRET for the surface-deposited QD-lipid nanoclusters observed by FLIM. An ETE of 52.6% was calculated from

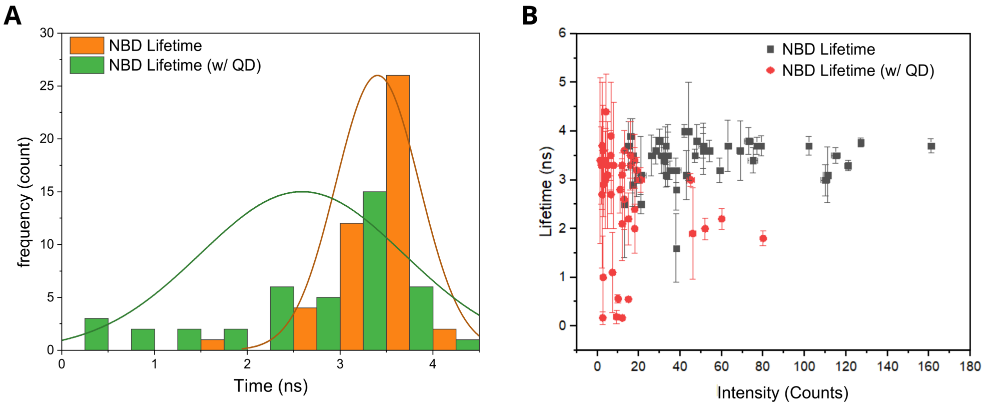

Figure S13: Distribution of fluorescence lifetimes and intensities of NBD obtained from FLIM. (A) Frequency distribution histogram of the fluorescence lifetime of NBD-DHPE in the control sample of liposomes without QDs (*orange*) and when co-localised with QDs in the sample of QD-lipid nanoclusters (*green*). (B) Scatter plot of fluorescence lifetime versus fluorescence intensity of NBD linked to lipids for the same samples as in (A): NBD signal for liposomes without QDs (*black*) and NBD signal co-localised with QDs for QD-lipid nanoclusters(*red*).

steady-state fluorescence measurements of the QD-lipid nanoclusters in solution, using the data in main text Figure 3D. These ETE values corresponds to a donor-to-acceptor distance of approximately 5 nm, indicating substantial lipid/QD co-localization, and thus, QD stability is maintained upon surface deposition. The fact that the ETE is lower once the QD nanoclusters associate with a surface could suggest that the some of the lipids associate tightly with the glass surface, forming a lipid bilayer, and possibly some fraction of the lipids disassociate with the QD nanoclusters during that process.

The NBD fluorescence lifetime versus intensity plot (**Figure S13B**) shows the differences due to the presence or absence of QDs in a different way to the histogram: each datapoint represent one single particle therefore the population distribution can be observed in this type of analysis. There is no clear correlation between the intensity and lifetime of NBD fluorescence for either situation, but we can observe a clear shift in the population. For the lipid-only sample (*black datapoints* in **Figure S13B**) there is great variability in the intensity from 10 to 150 counts but that the lifetime is relatively consistent at between 3 and 4 ns (with just a small number of outlier particles). Whereas, for the QD+lipid nanocluster sample (*red datapoints* in **Figure S13B**) there is population shift so that the majority of particles have a much lower NBD fluorescence intensity of 0-20 counts. The spread of NBD lifetimes is much greater in the sample with QDs than the sample without, ranging from 0-4 ns, including several particles with an NBD lifetime of 2-3 ns and several particles with lifetime 0-1 ns. The observation of very low NBD lifetime for a sub-population, due to the presence of QDs, suggests that some some NBD is very close to QDs, for example a QD that is in direct contact with a layer of lipid. The variability and higher NBD lifetimes in this same sample suggests that some lipids are far away from QDs, so there may be some "empty" lipid vesicles that are not associated with QDs.

Fitting of fluorescence decay curves

Fluorescence decay curves from the samples set where lipid:QD ratio was varied, main text **Figure 5**, were fit to a tri-exponential decay function. The fit parameters are shown in **Figure S14**.

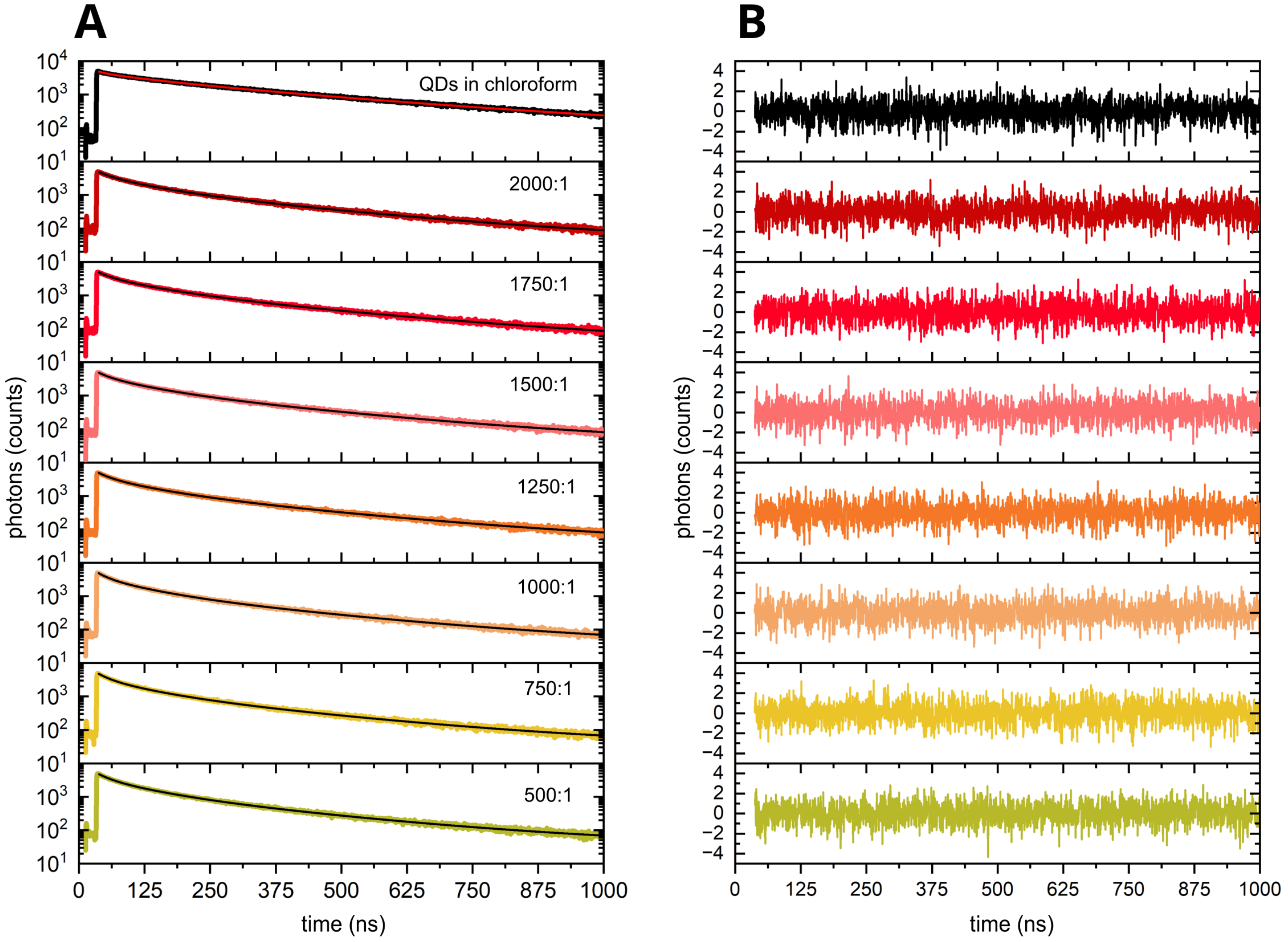

Sample	tau1	A1	tau2	A2	tau3	A3	chi^2	<τ> amp.	<τ> int.
CIS/ZnS QDs in Chloroform	16.9	0.13	109.6	0.28	339.5	0.59	1.04	**232.8**	305.8
500:1 lipid:QD Nanoclusters	24.0	0.29	86.0	0.43	263.1	0.28	1.02	**117.9**	193.1
750:1 lipid:QD Nanoclusters	21.5	0.29	79.8	0.43	261.6	0.28	1.05	**114.2**	193.9
1000:1 lipid:QD Nanoclusters	20.2	0.28	80.0	0.44	267.2	0.27	1.02	**114.4**	197.0
1250:1 lipid:QD Nanoclusters	24.0	0.28	90.6	0.43	286.7	0.28	0.98	**127.8**	212.5
1500:1 lipid:QD Nanoclusters	20.5	0.23	83.1	0.45	273.7	0.32	0.98	**128.7**	208.6
1750:1 lipid:QD Nanoclusters	24.0	0.27	94.9	0.44	295.0	0.29	1.04	**134.6**	220.0
2000:1 lipid:QD Nanoclusters	24.6	0.23	86.6	0.45	280.4	0.32	0.98	**134.3**	213.8

Figure S14: Fluorescence decay curve analysis of all samples with varying lipid:QD ratio including multi-component fit parameters. (A) Fluorescence decay curves from the samples referred to in main text Fig. 5. Raw data is shown in colour and the multi-exponential fit is overlaid as a solid line. The lipid ratio is labelled in each plot. (B) Residuals from the fits shown in panel A. *Below*, the table provides the full numerical parameters from each fit. Each curve was fit to a tri-exponential decay function, where A1, A2, A3 are the amplitudes corresponding to the lifetimes tau1, tau2, tau3, respectively. The corresponding amplitude-weighted and intensity-weighted mean lifetime values are shown, as tau $_{\text{amp.}}$ and tau $_{\text{int.}}$, respectively. The goodness of fit parameter chi^2 is shown.

Photophysics of Quantum Dot Nanoclusters

Fluorescence excitation versus emission measurements were conducted to explore potential alterations in energy transfer pathways that may occur for different arrangements of the $CuInS_2/ZnS$ QDs. We compared the colloidal form of the QDs that exists when the nanoparticles are dispersed in chloroform to the "nanoclusters" of QDs that form after the assembly procedure involving lipids and detergent as detailed in the main text. **Figure S15A** shows a 2-D contour plot that illustrates fluorescence excitation versus emission wavelengths for colloidal QDs, with maximum fluorescence intensity observed at an emission wavelength of 655 nm, when using excitation wavelengths of 350 to 600 nm. There is a shift of the fluorescence emission peak wavelength towards the red as the excitation wavelength approaches the emission peak (above 600 nm), denoted by the *black* arrows, indicating that there is a selective excitation of a lower-energy sub-population of the QDs when higher-wavelength excitation is used, resulting in relatively lower energy (higher wavelength) emission. This makes logical sense, because energy cannot be "gained" during the process. **Figure S15B** displays a 2-D contour plot of fluorescence excitation versus emission wavelengths for the QD-lipid nanoclusters, revealing a consistent maximum intensity at an emission wavelength of 700 nm with minimal shift in peak position for all excitation wavelength 350 to 650 nm. Again, there is a a red-shift of the fluorescence emission peak wavelength as the excitation wavelength approaches the emission peak (above 650 nm), again, as denoted by the black arrows.

Figure S15C shows the peak shifts relating to the data from **Figure S15A-B** even more clearly. This plot of the wavelength of fluorescence emission peak (maxima) against the wavelength of the excitation reveal a consistent trend for QD-lipid nanoclusters (*red*), consistently exhibiting an emission peak at 700 nm. This consistent emission peak suggests uniform energy transfer pathways within the QD clusters, irrespective of excitation energy. Notably, the emission peak remains stable until the excitation wavelength approaches 650-700 nm. In contrast, colloidal QDs (*black*) demonstrate a shift in emission towards lower-energy QDs as the excitation wavelength nears 600 nm. Overall, the findings from these excitation versus emission maps indicate a lack of excitation dependence on QD emission until nearing the emission peak, regardless of whether the QDs are arranged into clusters or not. This suggests that similar energy transitions occur within the system, leading to fluorescence from the same point regardless of the entry point of energy. Rather, the distinctive aspect for the nanoclusters lies in their enhanced connectivity, facilitating QD-to-QD transfers, which on average results in emission from QDs with smaller band gaps (lower energy, i.e., higher wavelength).

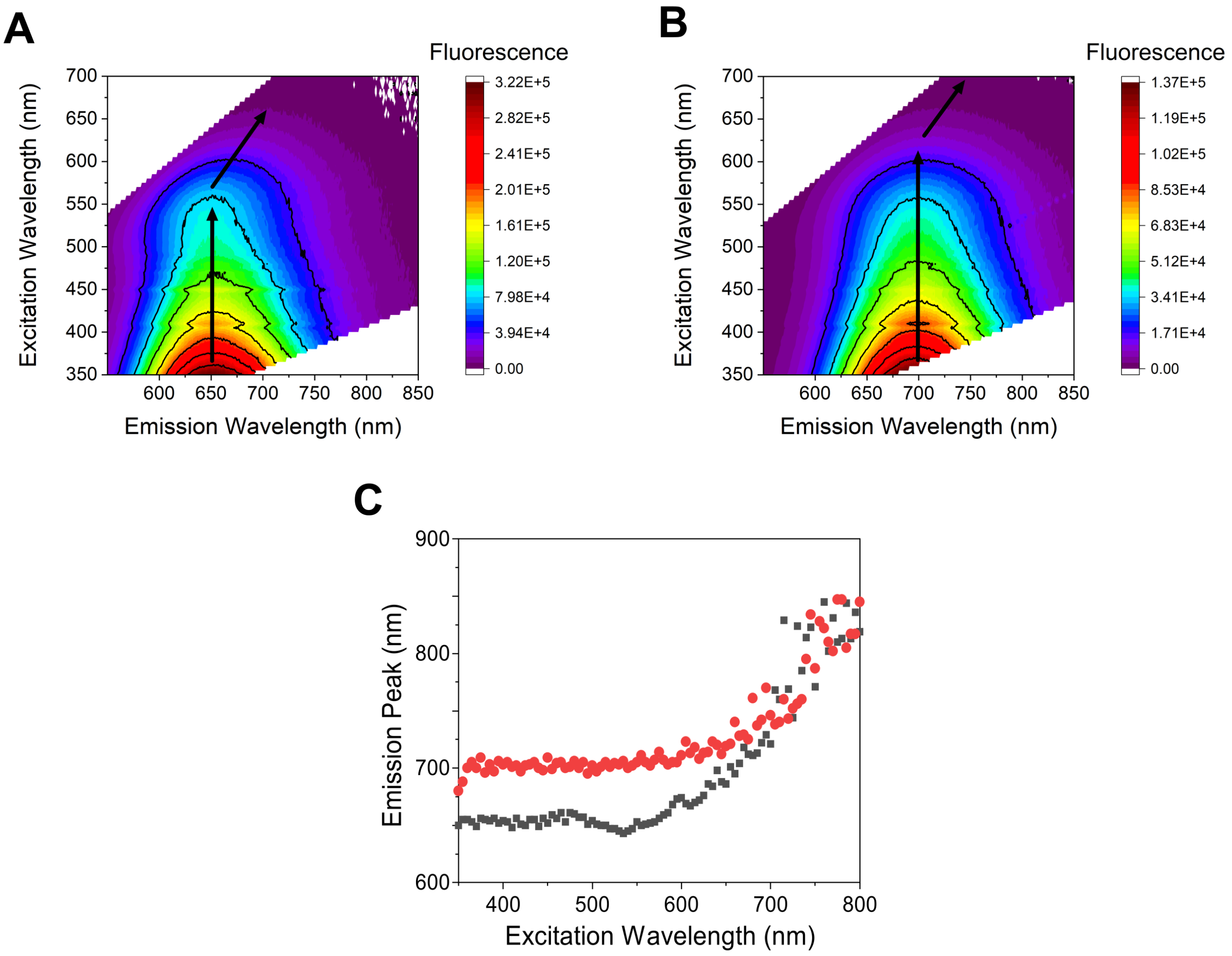

Figure S15: Fluorescence excitation versus emission plots comparing colloidal $CuInS_2$/ZnS QDs and nanoclustered $CuInS_2$/ZnS QDs. (A) Contour plot showing fluorescence intensity (colour-scale) as a function of fluorescence excitation wavelength and fluorescence emission wavelength for colloidal QDs in chloroform. (B) Contour plot showing fluorescence intensity as a function of excitation wavelength and emission wavelength for QD-lipid nanoclusters in aqueous buffer (50 mM HEPES, 100 mM NaCl, pH 7.5). (C) A scatter plot showing how the wavelength representing the centre of the fluorescence emission peak changes as the excitation wavelength is changed, for colloidal QDs in chloroform (*black*) and for QD-lipid nanoclusters in aqueous buffer (*red*).

References

[1] M. Booth, A. P. Brown, S. D. Evans and K. Critchley, *Chem. Mater.*, 2012, **24**, 2064–2070.

[2] A. J. Harvie, M. Booth, R. L. Chantry, N. Hondow, D. M. Kepaptsoglou, Q. M. Ramasse, S. D. Evans and K. Critchley, *Nanoscale*, 2016, **8**, 16157–16161.

[3] J. Kolny-Olesiak and H. Weller, *ACS Appl. Mater. Interfaces*, 2013, **5**, 12221–12237.

[4] L. Li, A. Pandey, D. J. Werder, B. P. Khanal, J. M. Pietryga and V. I. Klimov, *J. Am. Chem. Soc.*, 2011, **133**, 1176–1179.
